# Chitinase-3-like 1 regulates T_H_2 cells, T_FH_ cells and IgE responses to helminth infection

**DOI:** 10.3389/fimmu.2023.1158493

**Published:** 2023-07-27

**Authors:** Miranda L. Curtiss, Alexander F. Rosenberg, Christopher D. Scharer, Betty Mousseau, Natalia A. Ballesteros Benavides, John E. Bradley, Beatriz León, Chad Steele, Troy D. Randall, Frances E. Lund

**Affiliations:** ^1^ Department of Medicine, Division of Pulmonary, Allergy and Critical Care, University of Alabama Birmingham (UAB), Birmingham, AL, United States; ^2^ Department of Medicine, Section of Allergy and Immunology, Birmingham VA Medical Center, Birmingham, AL, United States; ^3^ Department of Microbiology, University of Alabama Birmingham (UAB), Birmingham, AL, United States; ^4^ Informatics Institute, University of Alabama at Birmingham, Birmingham, AL, United States; ^5^ Department of Microbiology and Immunology, Emory University, Atlanta, GA, United States; ^6^ Department of Medicine, Division of Rheumatology, University of Alabama Birmingham (UAB), Birmingham, AL, United States; ^7^ Department of Microbiology and Immunology, Tulane University, New Orleans, LA, United States

**Keywords:** IgE (immunoglobulin E), helminth, germinal center, T follicular helper (T cell FH, T helper 2 (Th2) cells, enteric, IL-4, germinal center (GC) B cells

## Abstract

**Introduction:**

Data from patient cohorts and mouse models of atopic dermatitis, food allergy and asthma strongly support a role for chitinase-3-like-1 protein (CHI3L1) in allergic disease.

**Methods:**

To address whether Chi3l1 also contributes to T_H_2 responses following nematode infection, we infected *Chi3l1* ^-/-^ mice with *Heligmosomoides polygyrus* (*Hp*) and analyzed T cell responses.

**Results:**

As anticipated, we observed impaired T_H_2 responses in *Hp*-infected *Chi3l1* ^-/-^ mice. However, we also found that T cell intrinsic expression of *Chi3l1* was required for ICOS upregulation following activation of naïve CD4 T cells and was necessary for the development of the IL-4^+^ T_FH_ subset, which supports germinal center B cell reactions and IgE responses. We also observed roles for *Chi3l1* in T_FH_, germinal center B cell, and IgE responses to alum-adjuvanted vaccination. While *Chi3l1* was critical for IgE humoral responses it was not required for vaccine or infection-induced IgG1 responses.

**Discussion:**

These results suggest that *Chi3l1* modulates IgE responses, which are known to be highly dependent on IL-4-producing T_FH_ cells.

## Introduction

Allergens are broadly defined as non-pathogenic proteins that induce specific IgE in sensitized individuals (1). However, sensitized individuals contact allergenic proteins in the context of a complex molecular milieu derived from living organisms. For example, feces of house dust mite (HDM) and cockroach contain major allergens complexed to lipid-binding or chitin-binding proteins, and protease activity is a well-known feature of HDM allergens, mold-derived allergens and the industrial sensitizer papain (2). Thus, individuals are typically sensitized to allergens in the context of inflammatory stimuli that provide adjuvant-like effects. Chitins, which are polysaccharides found within the exoskeleton of arthropods, crustaceans and helminths, and the cell wall of bacteria and fungi (3, 4), are thought to promote T_H_2 allergic responses (5, 6). Chitinases, which degrade chitins, and chitinase-like binding proteins (CLP), which can bind and sequester chitin but cannot degrade chitin (7), are upregulated in the lungs of asthma patients and allergen-exposed mice (7–9). Chitinase deficient mice (*Chit1* ^-/-^ or *Chia* ^-/-^) manifest enhanced airway inflammation in a HDM allergic airway disease model (10, 11), suggesting that chitinases function to attenuate allergic responses. In contrast, mice deficient in the CLP Chitinase-3-like 1 (*Chi3l1* ^-/-^) make impaired allergic airway responses following HDM exposure (8, 9) and mice deficient in Chitinase-like protein 3 (*Ym1*) develop reduced lung eosinophilia and mucus production in an ovalbumin model of allergic airway disease (12). This suggests that CLPs may not prevent allergic responses by sequestering chitin but rather function to facilitate allergic responses.

*CHI3L1* has been studied extensively in the setting of human allergic disease. Levels of YKL-40 (the protein product of the human *CHI3L1* gene) and expression of *CHI3L1* are increased in the lungs and serum of some asthma patient cohorts (13, 14). *CHI3L1* SNPs are reported to confer risk of asthma development and airway remodeling (14–17) as well as increased serum IgE and atopy (18) in patient cohorts. Moreover, increased expression of YKL-40 and *CHI3L1* have been linked to pathogenesis of allergic rhinitis (19, 20), atopic dermatitis (21–23) and food allergy (24). In agreement with human patient studies, experiments using *Chi3l1* ^-/-^ mice reveal that *Chi3l1* regulates type 2 cytokines and IgE levels in mouse models of asthma, atopic dermatitis and food allergy (8, 23–26). Thus, both mouse and human data support a role for YKL-40/Chi3l1 in promoting atopy and allergic disease.

Parasitic infections are recognized as the likely impetus for the evolution of T_H_2 immunity in mammals, and studies of parasitic infections demonstrate that mediators of protective immunity to parasitic infections often contribute to pathologic responses to allergens (27–30). *Chi3l1* ^-/-^ mice, which make attenuated allergic responses (8, 23–26), did not manifest impaired clearance of the nematode *Nippostrongylus brasiliensis* (*Nb*) during early neutrophil-mediated stages of infection, in spite of reduced lung IL-17A levels (31). Since the *Nb* study did not measure T_H_2 or IgE responses following *Nb* infection, we used the helminth *Heligmosomoides polygyrus bakerii* (*Hp*) to address whether *Chi3l1* is required for type 2 immunity in the setting of a strong T_H_2-driven enteric helminth infection. *Hp* primes a T_H_2 response (29, 32) and elicits IL-4 producing T_FH_ cells (33–36) that initiate polyclonal IgE production by B cells (35, 37). Here, we show that, consistent with the prior allergy studies, Chi3l1 regulates the IL-4^+^IL-13^+^ T_H_2 response to *Hp* infection. In addition, we observed that Chi3l1 controls the size of the T_FH_ compartment following both *Hp* infection and protein immunization. We demonstrate that *Chi3l1* ^-/-^ T_FH_ cells express significantly lower levels of Inducible T cell costimulator (ICOS) – a key regulator of T_FH_ development and maintenance in the B cell follicle (38, 39). RNA-seq analysis of the *Chi3l1* ^-/-^ T_FH_ cells revealed that these cells express the canonical T_FH_ transcriptional program but failed to acquire the normal transcriptional signature of IL-4 producing T_FH_ cells (40). These deficits were associated with decreased IL-4 production by the remaining *Chi3l1* ^-/-^ T_FH_ cells, decreased germinal center B cell responses and significantly decreased IgE^+^ ASCs and serum IgE following *Hp* infection or alum-adjuvanted vaccination. Thus, *Chi3l1* plays a nonredundant, unexpected and critical role in promoting IL-4^+^ T_FH_ cells that are known to amplify IgE antibody responses to both pathogens and allergens.

## Methods

### Mice

Animals were bred and maintained in the UAB animal facilities. All procedures were approved by the UAB IACUC and were conducted in accordance with the principles outlined by the National Research Council. BALB/cByJ mice (WT) and BALB/c CD45.1^+^ congenics (CByJ.SJL(B6)-Ptprc^a^/J) were purchased from The Jackson Laboratory. BALB/c BRP-39 (*Chi3l1* ^-/-^) mice (8) were provided by Dr. Allison Humbles (MedImmune). Bone marrow (BM) chimeras were generated by irradiating recipients with 850 Rads from a high-energy X-ray source (split dose 5 hours apart), and then reconstituting the recipients with 5x10^6^ total BM cells delivered *i.v.* To generate 50:50 chimeras we reconstituted irradiated recipients (BALB/cByJ) with 2.5x10^6^ BALB/c CD45.1^+^ (CByJ.SJL(B6)-Ptprc^a^/J) + 2.5x10^6^ *Chi3l1* ^-/-^ BM cells. Chimeras were used in experiments 8-12 weeks post-reconstitution. Both male and female mice were used and animals were matched for age and sex within an experiment. No gender-specific differences were observed.

### Heligmosomoides polygyrus stock

*H polygyrus* (*Hp*) stocks were maintained as described previously (33, 34). Mice were gavaged with 200 *Hp* L3 larvae.

### Flow cytometry

Mice were sacrificed at 8, 14 or 25 days post-infection (*p.i.*) for analysis of mesenteric lymph node (msLN) cells. Single cell suspensions from msLN, spleens or bone marrow (BM) were preincubated with FcR blocking antibody (2.4G2) after lysing red blood cells of spleens and BM with ACK lysis buffer (0.15 M NH4Cl, 10 mM KHCO3, 0.1 mM EDTA), then stained with cocktails of labeled antibodies. Antibody clone names and vendor include the following. Antibodies from BD: CD3 (17A2), CD4 (GK1.5), CD11b (M1/70), CD25 (PC61 BD), CD21 (7G6), CD43 (S7), CD44 (IM7), CD45.1 (A20), CD45.2 (104), B220 (RA3-6B2), CD62L (MEL-14), CD138 (281-2). CXCR5 (2G8), Bcl-6 (K112-91), BP1 (63C), Ly-6G (1A8) and IgE (R35-72). Antibodies from eBioscience: CD19 (1D3), CD23 (MCD2305), CD24 (M1/69), CD38 (90), CD93 (AA4.1), CD279/PD-1 (J43), CD278/ICOS (7E.17G9 and C398.4A), Foxp3 (FJK-16s, IgD (11-26), IgM (II/41) and B220 (RA3-6B2). Antibodies from BioLegend: CD4 (GK1.5), CD49b (DX5), CD150/SLAMF1 (TC15-12F12.2), and Ly-76 (TER-119). Other reagents included: AF488-labeled PNA (Life Technologies) or PNA (Sigma) labeled with Pacific Blue (Invitrogen), 7AAD (Invitrogen™), and LIVE/DEAD^®^ aqua or red (Life Technologies). Intracellular Bcl-6 and Foxp3 were detected using eBioscience™ Foxp3/Transcription Factor Staining Buffer set (Invitrogen™). To detect intracellular cytokines, cells were restimulated with 2.5 μg/mL plate-bound anti-CD3 (145-2C11, BioXcell) or 5 ng/mL PMA (Sigma) with 1.25 μM calcimycin (CalBiochem) plus Brefeldin A (BFA, 12.5 μg/mL, Sigma) for 4 hours. Cells were washed, incubated with FcBlock, surface stained and washed. Cells were fixed in formalin, permeabilized with 0.1% NP-40, washed, incubated with anti-cytokine antibodies (IL-4 (11B11, BD and Invitrogen), IL-13 (eBio13A, eBioscience), IFNγ (XMG1.2 eBioscience) and IL-17A (TC11-18H10, BD Pharmingen) in permeabilization buffer and washed. All incubations prior to fixation were performed with BFA. All flow analysis was performed using the BD Canto.

### *In vitro* activation of CD4 T cells

CD4^+^ T cells were purified from spleens of uninfected BALB/c CD45.1^+^ (CByJ.SJL(B6)-Ptprc^a^/J), BALB/c and *Chi3l1* ^-/-^ mice using CD4 MACS L3T4 beads (Miltenyi Biotech) and stimulated for 48 hours as previously described (41) with 2.5 μg/mL plate-bound anti-CD3 (145-2C11, BioXcell) and 2.5μg/ml plate-bound anti-CD28 (37.51, eBioscience). To mimic early T_FH_ development cells were cultured in the presence of 10 μg/ml rIL-6 (R&D) and neutralizing antibodies to IL-2 (JES6-1A12, BioXcell, 5 μg/ml and S4B6-1 BioXcell, 5 μg/mL). For CD4 T cell co-cultures, BALB/c or *Chi3l* ^-/-^ CD45.2^+^ T cells were mixed at a 1:1 ratio with CD45.1^+^ T cells and then stimulated for 48 hours with CD3 and CD28 antibodies.

### Cell sorting

14 days after oral gavage with 200 L3 *Hp* (D14), infected BALB/c ByJ and *Chi3l1* ^-/-^ mice were sacrificed and msLN were collected and pooled. Single cell suspensions of msLN cells were incubated with FcBlock (BD), enriched with CD4 microbeads (Miltenyi), and stained with fluorochrome-conjugated antibodies. CD4^+^CXCR5^+^PD-1^hi^Lin^neg^ cells were sorted (BD Aria, UAB Flow Cytometry Core), pelleted, lysed in TRIzol (ThermoFisher), and stored at -80°C until used.

### Production of recombinant influenza NS1 antigen and NS1 tetramers

The influenza NS1 gene, modified to contain a 3’ in frame addition of the BirA enzymatic biotinylation site and the 6X-His purification tag (GeneArt), was mutated at R38A and K41A (to prevent aggregation of NS1 at high concentrations (42)), then cloned into the pTRC–His2c expression vector (Invitrogen) and expressed in the BirA-enzyme containing *E. coli* strain CVB101 (Avidity). Biotinylated recombinant NS1 was purified by FPLC and then tetramerized to fluorochrome-conjugated streptavidin (Prozyme). To detect NS1-specific B cells, PE-labeled NS1 tetramers (1:100) were incubated with cells for 30 min at 4°C.

### NP(15)-OVA or NS1 immunization

50 μg biotinylated NS1 protein or 50 μg (4-hydroxy-3-nitrophenyl)-acetyl(15)-OVA (NP-OVA) was diluted to 1 mg/mL in PBS and adsorbed to 100 μg alhydrogel alum (InVivoGen) in a total of 200 μL per mouse for 30 min at room temperature, then injected *i.p.* on day 1 and analyzed on day 12 (D12) (splenic T and B cell responses) or day 14 (D14) (serum antibody).

### ELISAs

#### IgG1 detection

Serum from uninfected mice was analyzed for total IgG1 using a mouse clonotyping kit and standards (Southern Biotech) according to manufacturer’s recommendations. *Hp-*specific IgG1 was detected as previously described (43) using plates coated with *Hp* extract and detected using rat anti-mouse IgG1-HRP antibody (Southern Biotech). NP-OVA specific IgG1 was detected as previously described (44) using plates coated with NP(5)-BSA and detected using goat anti-mouse IgG1-HRP (Southern Biotech).

#### IgE detection

For analysis of total IgE in serum from uninfected, D21 *Hp*-infected mice, or NS1 immunized mice, ELISAs were performed using paired rat anti-mouse IgE antibodies (capture antibody: 1 μg/mL purified R35-72, detect antibody biotinylated R35-118, BD) and streptavidin-HRP (BD). Purified mouse IgE K was used as a standard (C38-2, BD). To detect NP-OVA specific IgE, plates were coated with 1 μg/mL purified anti-IgE (R35-72, BD). Samples were applied by 2-fold dilutions to the coated plates. Specific IgE was detected by biotinylated NP(5)-BSA followed by streptavidin-HRP.

### RNA-seq analysis

RNA was isolated (RNeasy micro column (Qiagen)) from T_FH_ cells sorted into TRIzol as previously described (45) and then enriched with Oligo(dT) beads. Sequencing libraries (NEBNext Ultra II RNA Library Prep Kit for Illumina (NEB, Ipswitch, MA, USA)) were prepared by GeneWiz LLC (South Plainfield, NJ, USA) and sequenced with a 2x150bp Paired End configuration on an Illumina HiSeq 4000. Image analysis and base calling were conducted using Hiseq Control Software (HCS). Raw sequence data (.bcl files) generated from Illumina HiSeq was converted into fastq files and de-multiplexed using Illumina’s bcl2fastq 2.17 software. One mismatch was allowed for index sequence identification. Adapter content was removed from fastq files using Skewer 0.2.2 (46) and data aligned with STAR 2.5.3a (47) to the ENSEMBLE BALB/c/j GCA_001632525 reference mouse genome and transcriptome. PCR duplicate reads were flagged using PICARD MarkDupilicates 1.127 (http://broadinstitute.github.io/picard), gene counts for each sample were computed using GenomicRanges 1.34.0 (48), and reads per kilobase per million (RPKM) normalized in R 3.5.2. Genes with at least 3 reads per million (RPM) in all samples of either the WT and/or *Chi3l1* ^-/-^ T_FH_ groups were considered detected. After confirming that the first 8 exons of the *Chi3l1* gene, which were deleted in the *Chi3l1* ^-/-^ mouse strain, were not expressed in the *Chi3l1* ^-/-^ T_FH_ samples, we removed the *Chi3l1* gene (also called *Chil1*) from the RNA-seq analysis as exons 8-10 of the *Chi3l1* gene, which are directly downstream from the inserted promoter + neomycin cassette (8), were expressed, presumably due to the insertion of the neomycin deletion cassette.

9853 expressed genes (defined as genes with at least 3 reads per million (RPM) in all samples of either the WT and/or *Chi3l1* ^-/-^ T_FH_) were identified. Differential expression between groups was analyzed using DESeq2 1.26.0 (49) and 1465 genes were identified as significantly different between WT and *Chi3l1* ^-/-^ T_FH_ cells using an FDR cutoff of q<0.05. See **Table S1** for gene expression levels and detailed methods. 193 genes with average reads per kilobase of transcript per million (RPKM) > 1 in either group and meeting a criteria of FDR p < 0.05 and threshold of ±0.3785 log_2_ fold-change (FC) (1.3-fold) were submitted to Ingenuity Pathway Analysis (IPA, QIAGEN Digital Insights), of which 182 were used by IPA to identify significantly enriched pathways. For gene set enrichment analysis (GSEA), expressed genes from WT and *Chi31l* ^-/-^ T_FH_ cells were ranked by multiplying the -log_10_ of the P-value from DESeq2 (49) by the sign of the fold change and then used as input in the GSEA (50) PreRanked analysis program (http://software.broadinstitute.org/gsea/index.jsp). RNA-seq data sets were deposited in the NCBI Gene Expression Omnibus (GSE203113). RNA-seq processing code is available at https://github.com/cdschar/Curtiss_Tfh_RNAseq/.

### Statistical analysis

Statistical details of all experiments including tests used, n, and number of experimental repeats are provided in figure legends. FlowJo (version 9, Tree Star) was used for flow cytometric analyses. Prism Graphpad (version 9) was used for statistical analyses of flow cytometry experiments. Statistical analysis of RNA-seq experiments is summarized within the text of the RNA-seq experimental design in **Table S1**.

## Results

### *Chi3l1* regulates IL-4 production by restimulated LN CD4^+^ T effectors from *Hp*-infected mice

Murine allergic airway disease models revealed that type 2 cytokine responses are blunted in *Chi3l1* ^-/-^ animals (8) and that *Chi3l1* ^-/-^ CD4 T cells primed *in vitro* with antibodies to CD3 and CD28 in the presence of T_H_2-polarizing conditions are impaired in IL-4 production (51). To test whether *Chi3l1* regulates T_H_2 responses to helminth infection, we measured mesenteric LN (msLN) CD4 T cell responses in wild-type (WT) BALB/c and *Chi3l1* ^-/-^ BALB/c mice that were orally infected with *Hp*. Consistent with previous studies (51), uninfected *Chi3l1* ^-/-^ mice did not exhibit changes in the numbers of total msLN cells or CD19 B cells (**Figures S1A–C**). However, the msLN total CD4 and activated CD44^hi^CD62L^lo^ CD4 T cell responses were attenuated in *Hp*-infected *Chi3l1* ^-/-^ mice (**Figures S1D–F**). To determine whether the remaining *Chi3l1* ^-/-^ CD4 T cells were competent to produce T_H_2 cytokines, we analyzed intracellular cytokine levels in anti-CD3 restimulated msLN CD44^hi^ CD4 T cells from D8 *Hp*-infected mice. We observed that the percentage and number of CD44^hi^ *Chi3l1* ^-/-^ CD4 T_H_2 cells making both IL-4 and IL-13 were significantly decreased in *Chi3l1* ^-/-^ T cells relative to the restimulated WT T cells (**Figures S2A–C**). This impairment in type 2 cytokine production was not rescued with PMA+calcimycin stimulation (**Figures S2D–G**) and, consistent with the dominant T_H_2 response to *Hp* infection (52), few CD4 T cells from BALB/c or *Chi3l1* ^-/-^ mice produced IFNγ (**Figures S2H–J**) or IL-17A (**Figures S2K–M**) following anti-CD3 stimulation. These data therefore suggest that the development or effector potential of parasite-elicited CD4 T cells was compromised in *Chi3l1* ^-/-^ mice.

### *Chi3l1* regulates T_FH_ responses to *Hp* infection

**Figure 1:**
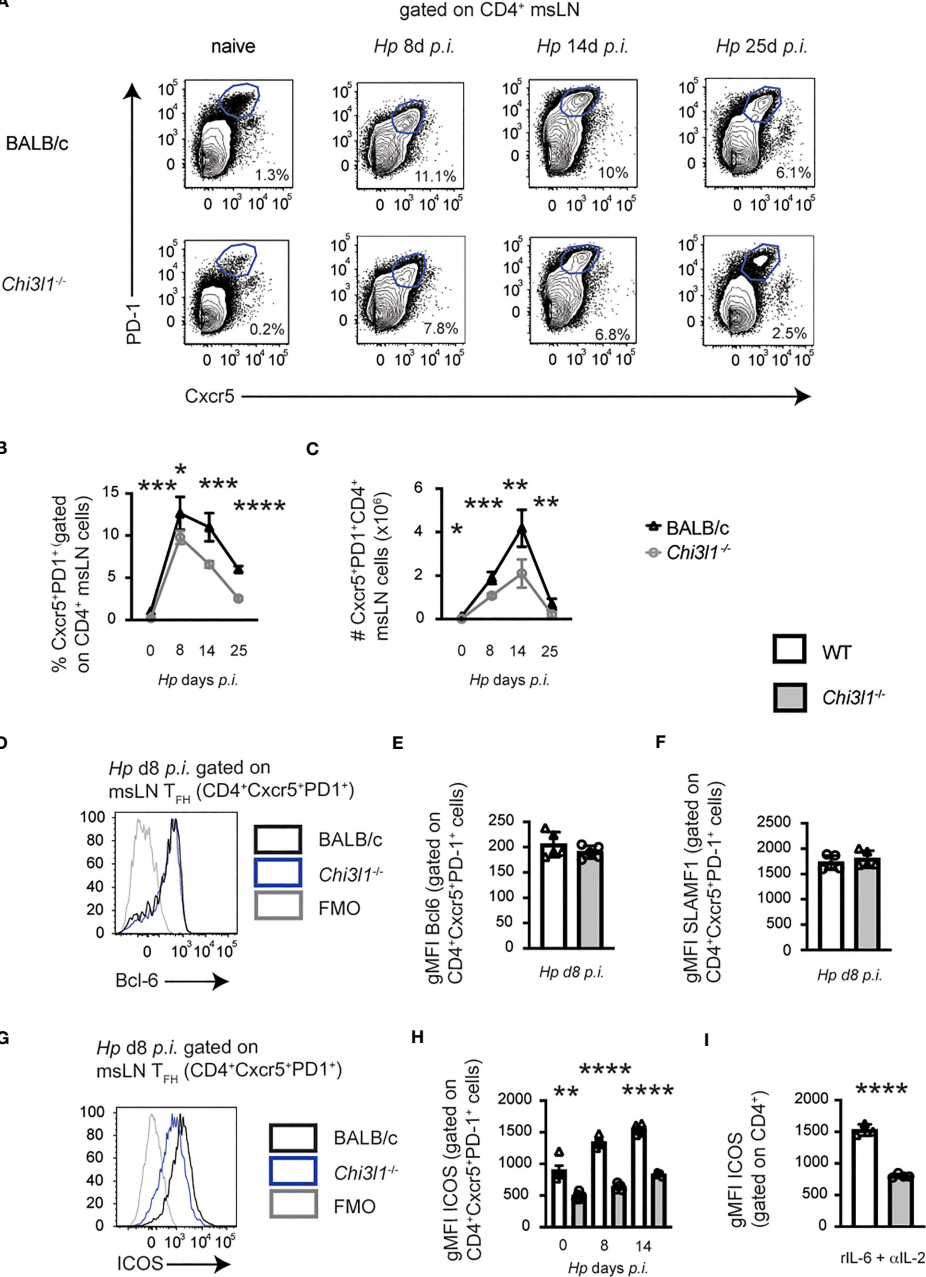
*Chi3l1* regulates T_FH_ responses to *Hp* infection. **(A–H)** Enumeration and characterization of msLN T_FH_ cells from uninfected (n=5/group) or *Hp*-infected BALB/c (white bars) and *Chi3l1* ^-/-^ (grey bars) mice (n=5/group). **(A–C)** T_FH_ response to *Hp* infection with flow plots **(A)** showing CXCR5^+^PD-1^+^ T_FH_ cells between D0 (uninfected) and D25 post-*Hp* with frequency **(B)** and number **(C)** of T_FH_ cells. Kinetics of the msLN response (enumeration of total msLN cells, CD19 B cells, CD4 T cell and activated CD44^hi^CD62L^lo^) shown in **Figures S1A–F**. Cytokine production by CD44^hi^ CD4 T cells shown in **Figure S2**. **(D–H)** T_FH_ phenotype in msLN of uninfected (D0) and *Hp*-infected mice. Bcl-6 **(D, E)**, SLAM **(F)** and ICOS **(G, H)** expression by T_FH_ on D8 **(D–G)** or between D0-D14 **(H)**. Flow cytometry plots for Bcl-6 **(D)** and ICOS **(G)** are shown. Expression levels of Bcl-6 **(E)**, SLAM **(F)** or ICOS **(G)** in T_FH_ cells presented as the geometric Mean Fluorescence Intensity (gMFI). **(I)** gMFI of ICOS expression by purified splenic CD4^+^ T cells from uninfected BALB/c (white bars) and *Chi3l1* ^-/-^ (grey bars) mice (n=4/group) that were stimulated *in vitro* for 48 hours in triplicate with anti-CD3 plus anti-CD28 in the presence of rIL-6 plus anti-IL-2. Data representative of 2 **(D–F)** or ≥ 3 independent experiments (all others). **(A–H)** Data displayed as the mean ± SD of each group with individual animals depicted as circles or triangles. **(I)** Data displayed as the mean ± SD of each group with individual animals (assayed in triplicate) depicted as circles or triangles. Statistical significance determined using unpaired 2-tailed student’s t test. *p≤0.05, **p≤0.01, ***p≤0.001, ****p≤0.0001.

Helminth infections induce robust T follicular helper (T_FH_) responses (33, 53). Since T_FH_ responses have not been analyzed in *Chi3l1* ^-/-^ mice, we enumerated T_FH_ (CXCR5^+^PD-1^hi^) cells in the msLN of uninfected and *Hp*-infected animals. T_FH_ cells (**Figure 1A**) were detected at low levels in the msLN of uninfected BALB/c mice and increased in both frequency and number (**Figures 1B, C**) over the first two weeks following *Hp* infection. Although the kinetics of the T_FH_ responses were similar between the *Hp*-infected BALB/c and *Chi3l1* ^-/-^ mice, the magnitude of the *Chi3l1* ^-/-^ T_FH_ response was significantly decreased (**Figures 1B, C**). Moreover, while the T_FH_ master regulator (54) Bcl-6 (**Figures 1D, E**) and T_FH_-supporting receptor SLAMF1 (CD150) (55) (**Figure 1F**) were expressed at similar levels in the msLN BALB/c and *Chi3l1* ^-/-^ T_FH_ cells, expression of ICOS, a key co-stimulatory molecule for T_FH_ cells (39, 54, 56–58), was significantly decreased on the *Chi3l1* ^-/-^ CXCR5^+^PD-1^hi^ T_FH_ cells throughout infection (**Figures 1G, H**). To assess whether the reduction in ICOS expression in *Chi3l1* ^-/-^ T_FH_ cells was likely to be cell intrinsic, we activated splenic CD4 T cells from uninfected WT and *Chi3l1* ^-/-^ mice under early T_FH_ differentiation conditions (41) with platebound anti-CD3 + anti-CD28 and IL-6 plus IL-2 blocking antibodies. We observed decreased ICOS upregulation in the *in vitro* activated *Chi3l1* ^-/-^ CD4 T cells (**Figure 1I**). Together, these data suggest that *Chi3l1* does not control T cell commitment to the T_FH_ lineage but might be important for the expansion, maintenance, or function of this T cell subset.

### ICOS expression by Foxp3^+^CD25^+^ T_REG_ does not require *Chi3l1*

Since ICOS levels were decreased in the *Chi3l1* ^-/-^ T_FH_ cells, we asked whether ICOS expression was altered in Foxp3^+^CD25^+^CD4^+^ cells, since ICOS function is critical in both T_FH_ and T_REG_ development during *Hp* infection (59). We examined mice on D25 post *Hp*-infection – a timepoint when the CD4 T cell response is contracting and T_REG_ responses are induced to facilitate establishment of a chronic infection (29, 60, 61). The numbers of CD25^+^Foxp3^+^ T_REG_ (**Figures S1G–H**) were similar between the groups. Moreover, we did not observe reduced ICOS expression by *Chi3l1* ^-/-^ CD25^+^Foxp3^+^ T_REG_s (**Figures S1I–J**). Thus, *Chi3l1* modulates ICOS expression by *Hp*-infection elicited T_FH_ cells but not T_REG_ cells.

### Hematopoietic cell expression of *Chi3l1* regulates T_H_2 and T_FH_ responses to *Hp*

Transgene-directed *Chi3l1* expression by epithelial cells is reported to restore lung T_H_2 cytokine levels in allergen-exposed *Chi3l1* ^-/-^ mice (8). To assess whether the attenuated T_H_2 and T_FH_ responses to *Hp* were due to *Chi3l1* expression by radiation resistant cells, like epithelial cells, or to *Chi3l1* expression by radiation sensitive cells, like bone marrow (BM)-derived immune cells, we analyzed *Hp*-elicited T cell responses in BALB/c and *Chi3l1* ^-/-^ mice that were lethally irradiated and reconstituted with either BALB/c BM or *Chi3l1* ^-/-^ BM. msLN cells of BM chimeras that were competent to express *Chi3l1* in all cell types (WT donor/WT recipient), lacked *Chi3l1* in all cell types (KO donor/KO recipient), lacked expression of *Chi3l1* specifically in the hematopoietic compartment (KO donor/WT recipient) or lacked expression of *Chi3l1* in the radiation resistant compartment (WT donor/KO recipient) were compared 8 days after *Hp* infection. We found no statistically significant differences in total numbers of msLN cells, B cells, or CD4 T cells in any of the groups (**Figures S3A–C**). However, and consistent with our analysis of non-chimeric *Chi3l1* ^-/-^ mice, we observed a significant reduction in the numbers of total activated CD62L^lo^CD44^hi^ CD4 cells (**Figure S3D**) in the KO donor/KO recipient mice compared to the WT donor/WT recipient mice. Similarly, the day 8 *Hp*-infected KO donor/KO recipient mice had decreased frequencies and numbers of CXCR5^+^PD-1^+^ T_FH_ cells (**Figures S3E–G**), which expressed lower levels of ICOS (**Figure S3H**). Moreover, the T cells from the KO donor/KO recipients produced significantly less IL-4 and IL-13 following anti-CD3 restimulation when compared to the T cells from the WT donor/WT recipients (**Figures S3I–K**). When we compared the data from these chimeras to the other two groups of chimeras, we found no differences in any of the T cell responses between the KO donor/WT recipients and the KO donor/KO recipients (**Figures S3D–K**). These results indicated that *Chi3l1* expression by BM-derived cells was necessary for optimal CD4 T cell responses to *Hp* infection. Consistent with this idea, the CD4 T cell responses were largely, although not completely, rescued (**Figures S3D–K**) in *Chi3l1* ^-/-^ mice reconstituted with WT BM (WT donor/KO recipient). Thus, while we could not completely rule out a role for Chi3l1 expressing non-hematopoietic cells in regulating CD4 T cell responses to *Hp*, our data indicated that CD4 T cell responses to *Hp* minimally require hematopoietic cell expression of *Chi3l1*.

**Figure 2:**
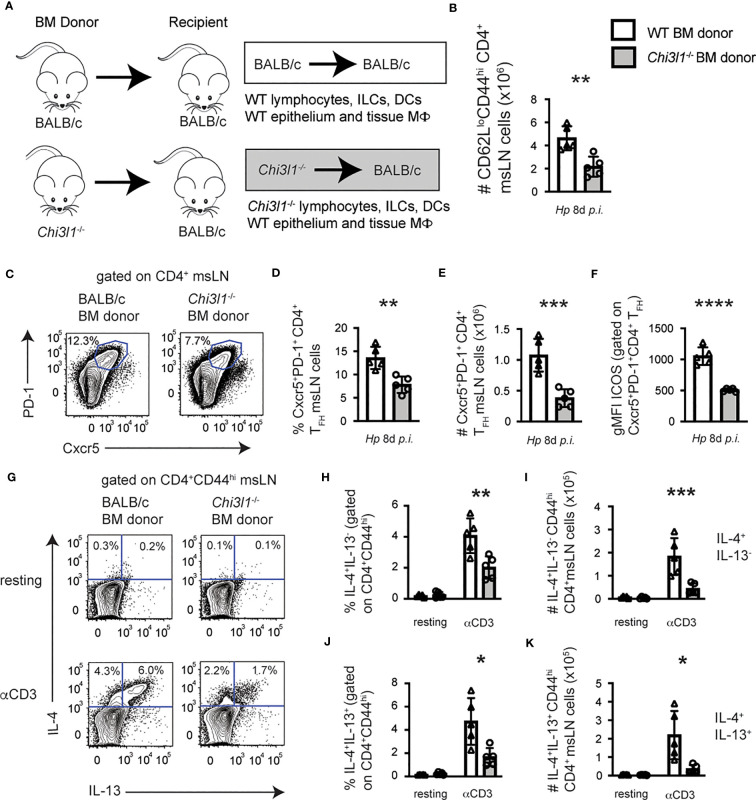
*Chi3l1* expressing hematopoietic cells are necessary and sufficient for T_FH_ and T_H_2 responses to *Hp*. Enumeration of T cell responses in msLNs of D8 *Hp*-infected BM chimeric mice (n=5 mice/group) that were generated **(A)** by reconstituting lethally-irradiated BALB/c recipient mice with BALB/c (white bars) or *Chi3l1* ^-/-^ (grey bars) BM. msLN CD4^+^ CD62L^lo^CD44^hi^ cells from D8 *Hp*-infected chimeric mice were analyzed directly *ex vivo* **(C–F)** or following restimulation for 4 hours with anti-CD3 in the presence of BFA **(G–K)**. **(B–E)** Number **(B)** of activated CD4 T cells in msLN from D8 *Hp*-infected mice. Representative flow cytometry plot **(C)** showing CXCR5^+^PD-1^+^ T_FH_ cells in D8 msLNs with the percentages **(D)** and numbers **(E)** of T_FH_ cells and ICOS expression levels **(F)** by T_FH_ cells represented as gMFI. **(G–K)** IL-4 and IL-13 production by resting and anti-CD3 stimulated msLN cells. Representative flow plot **(G)** showing IL-4 and IL-13 expression by CD44^hi^ CD4 cells from D8 *Hp*-infected chimeras with the percentage and number of IL-4^+^IL-13^neg^ **(H–I)** and IL-4^+^IL-13^+^ **(J–K)** producers. Analysis of T_FH_ responses and CD4 T cell responses in reciprocal BM chimeras is shown in **Figure S3**. Data representative of ≥ 3 independent experiments displayed as mean ± SD of each group with cells from individual animals depicted as circles or triangles. Unpaired 2-tailed student’s t-test was used to assess statistical significance. *p≤0.05, **p≤0.01, ***p≤0.001, ****p≤0.0001.

To further assess the contribution of *Chi3l1*-expressing hematopoietic cells to *Hp*-induced CD4 T cell responses, we directly compared *Hp*-elicited T cell responses in BALB/c and *Chi3l1* ^-/-^ mice that were lethally irradiated and reconstituted with either BALB/c BM or *Chi3l1* ^-/-^ BM (**Figure 2A**). We observed a significant reduction in the numbers of total activated CD62L^lo^CD44^hi^ CD4 cells (**Figure 2B**) and CXCR5^hi^PD-1^hi^ T_FH_ cells (**Figures 2C–E**) in the *Hp*-infected BALB/c recipients reconstituted with *Chi3l1* ^-/-^ BM relative to animals reconstituted with BALB/c BM. Expression of ICOS on CXCR5^+^PD-1^hi^ T_FH_ cells was reduced in mice reconstituted with *Chi3l1* ^-/-^ BM (**Figure 2F**). We confirmed the effector potential of CD4 T cells from BALB/c mice reconstituted with *Chi3l1* ^-/-^ BM chimeras was impaired, as the frequencies and numbers of IL-4^+^ single producers (**Figures 2G–I**) and IL-4^+^IL-13^+^ double producers (**Figures 2J, K**) were decreased following anti-CD3 stimulation. Thus, *Chi3l1* expression within BM derived radiation sensitive immune cells regulates ICOS expression by T_FH_ cells and is required for development of T_FH_ responses and T_H_2 cells with effector potential following *Hp* infection.

### Cell intrinsic expression of *Chi3l1* regulates B cell development but appears not critical for T_FH_ development or expansion

**Figure 3:**
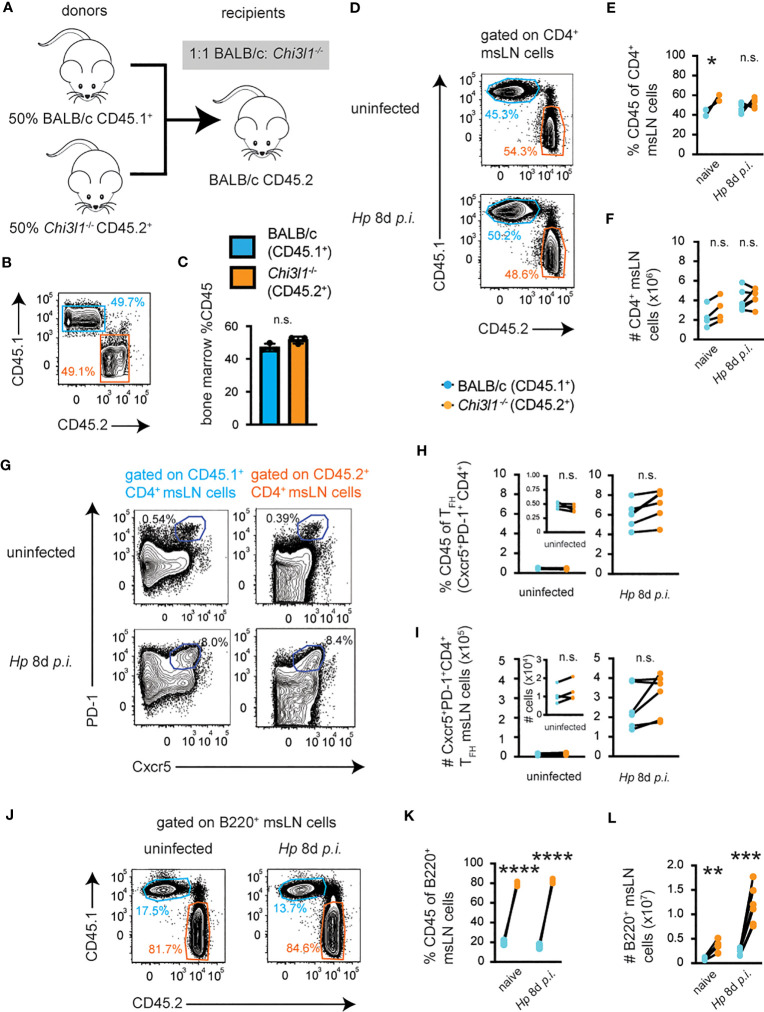
*Chi3l1* regulates T_FH_ development and expansion *via* a cell-extrinsic mechanism. Enumeration of T and B cell responses in mLNs from *Hp*-infected mixed 50:50 BM chimeras that were generated **(A)** by reconstituting lethally-irradiated BALB/c recipient mice with a 1:1 ratio **(B, C)** of BALB/c (CD45.1^+^, teal bars) and *Chi3l1* ^-/-^ (CD45.2^+^, orange bars) BM. Chimeras were analyzed before infection (n=4/group), on D8 (n=6/group) or on D14 (n=5/group) post-*Hp* infection. Cells were analyzed directly *ex vivo*. **(D–I)** Flow plots **(D)** showing BALB/c CD45.1^+^ (teal) and *Chi3l1* ^-/-^ CD45.2^+^ (orange) donor-derived msLN CD4 T cell populations before and on D8 post-infection. Frequency **(E)** and number **(F)** of CD4 cells of each genotype derived from each animal at each timepoint. Flow plots **(G)** showing both donor-derived T_FH_ populations in uninfected and D8 post-infection with frequency **(H)** and number **(I)** of T_FH_ cells of each genotype derived from each animal at each timepoint. **(J–L)** Flow plots **(J)** showing BALB/c CD45.1^+^ (teal) and *Chi3l1* ^-/-^ CD45.2^+^ (orange) donor-derived msLN B220^+^ B cell populations before and on D8 post-infection. Frequency **(K)** and number **(L)** of B220^+^ B cells of each genotype derived from each animal at each timepoint. Analysis of B cell development in BM and spleen in 50:50 chimeras is shown in **Figure S4**. Data representative of ≥3 independent experiments. Data displayed as mean ± SD in triplicate shown as bars **(C)** or as cell populations derived from each genotype from individual animals shown as paired lines **(E–L)**. Statistical analysis was performed with unpaired 2-tailed student’s t-test **(C)** or paired 2-tailed student’s t-test (all others). *p≤0.05, **p≤0.01, ***p≤0.001, ****p≤0.0001. *p≤0.05, **p≤0.01, ***p≤0.001, ****p≤0.0001.

CHI3L1 is a secreted protein (62, 63) and may regulate T cell responses to *Hp via* autocrine and/or paracrine mechanisms. To address whether T cell-intrinsic expression of *Chi3l1* regulates T_H_2 and T_FH_ responses to *Hp*, we measured CD4 T cell responses in *Hp*-infected BM chimeras (**Figure 3A**) that were reconstituted with a 1:1 mixture of BM derived from CD45.1^+^ WT BALB/c congenic animals and CD45.2^+^ *Chi3l1* ^-/-^ mice (**Figures 3B, C**). Although the percentage (**Figures 3D, E**) of *Chi3l1* ^-/-^ CD4 msLN cells in naïve chimeras was modestly increased relative to the BALB/c CD4 T cells present in the same animal, the numbers (**Figure 3F**) of WT and *Chi3l1* ^-/-^ CD4 T cells were similar in uninfected 50:50 chimeras. This was not limited to CD4 T cells in the msLN as the ratios of WT to *Chi3l1* ^-/-^ splenic CD4 T cells, CD8 T cells and myeloid cells (CD19^neg^CD3^neg^DX5^neg^) cells were similar (**Figures S4A–E**), even after correcting for BM input (**Figure S4F**). Moreover, the percentages (**Figures 3D, E**) and numbers (**Figure 3F**) of msLN *Chi3l1* ^-/-^ and BALB/c CD4 cells isolated from the same chimeric recipient on day 8 post-*Hp* infection were not significantly different. Similarly, we observed no significant differences in either the frequency (**Figures 3G, H**) or number (**Figure 3I**) of T_FH_ cells of each genotype present in same host on D0 or 8 days post-infection.

While T cell intrinsic expression of *Chi3l1* did not appear to be required for the development of the T_FH_ compartment, we did observe that the percentage (**Figures 3J, K**) and number (**Figure 3L**) of *Chi3l1* ^-/-^ B220^+^ msLN cells was significantly increased relative to BALB/c B220^+^ B cells in both uninfected and *Hp*-infected 50:50 chimeric mice. The same phenotype was also observed in the spleen (**Figures S4C–E**), even after normalizing for the input BM cells in the 50:50 chimeras (**Figure S4F**). To see if this effect was limited to mature B cells in the periphery, we examined B cell development in the BM of the 50:50 chimeras. We found that the BM of these chimeras was heavily skewed toward the *Chi3l1* ^-/-^ B cell progenitors at multiple stages of B cell development, including the pre-B cell stage (Fraction C-C’) in the BM (**Figures S4G–K**), and between Fraction E of the BM and development of mature follicular and marginal zone B cells in the spleen (**Figures S4L–N**). Since the number of mature B cells was not altered in non-chimeric *Chi3l1* ^-/-^ mice (**Figure S1C**), or in reciprocal BM chimeras (**Figure S3B**) we concluded that a B cell intrinsic role for *Chi3l1* during B cell development was revealed when WT and *Chi3l1* ^-/-^ B lineage precursors were forced to compete in the 50:50 BM chimeras.

### CD4 T cell-intrinsic *Chi3l1* is required for functional T cell responses to *Hp*

**Figure 4:**
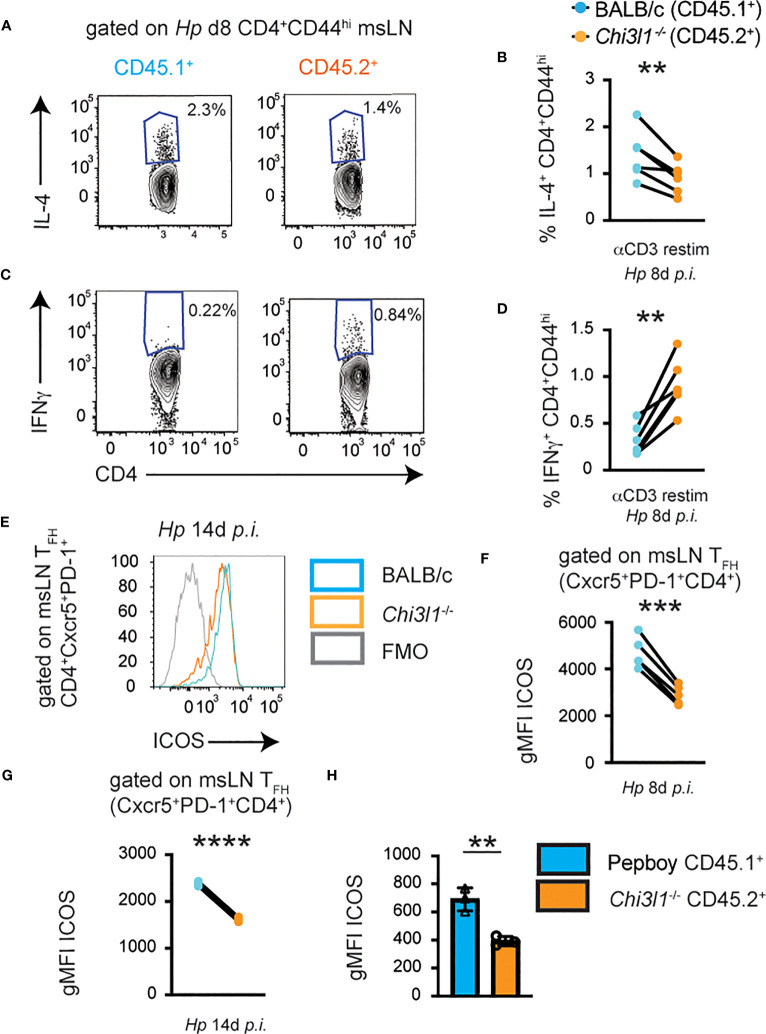
*Chi3l1* regulates cytokine production and ICOS expression in T cells by a cell intrinsic mechanism. **(A–G)** CD4 T responses in mLNs from *Hp*-infected mixed 50:50 BM chimeras that were generated as described in **Figure 3** with 50% BALB/c (CD45.1^+^) BM plus 50% *Chi3l1* ^-/-^ (CD45.2^+^) BM. **(A–D)** Cytokine production by anti-CD3 restimulated donor D8 BALB/c CD45.1^+^ (teal) and *Chi3l1* ^-/-^ CD45.2^+^ (orange) CD44^hi^ CD4 cells. Flow plots showing IL-4 **(A)** and IFNγ **(C)** production with the percentage of IL-4 **(B)** and IFNγ **(D)** producers of each genotype derived from each D8 *Hp*-infected animal. **(E–G)** ICOS expression by donor BALB/c CD45.1^+^ (teal) and *Chi3l1* ^-/-^ CD45.2^+^ (orange) CXCR5^+^PD-1^+^ T_FH_ cells on D8 and D14 post-*Hp* infection. Flow plots **(E)** showing ICOS expression by D14 T_FH_ cells. gMFI of ICOS expression levels on D8 **(F)** and D14 **(G)** post-infection by BALB/c or *Chi3l1* ^-/-^ donor-derived T_FH_ cells from the same animal. **(H)** gMFI of ICOS expression by purified splenic CD4^+^ T cells from uninfected BALB/c CD45.1^+^ mice (teal bars) that were co-cultured in triplicate at a 1:1 ratio with CD4 T cells from *Chi3l1* ^-/-^ CD45.2^+^ (orange bars) mice (n=3/group) and stimulated for 48 hours with anti-CD3 plus anti-CD28. Data representative of ≥3 **(A-G)** or 2 **(H)** independent experiments. Data in **(A–G)** represent cell populations derived from each genotype from individual animals shown as paired lines. Data in **(H)** displayed as the mean ± SD of each group with individual animals (assayed in triplicate) depicted as circles or triangles. Statistical analysis was performed with paired 2-tailed student’s t-test **(A–G)** or unpaired 2-tailed student’s t-test **(H)**. **p≤0.01, ***p≤0.001, ****p≤0.0001.

Although T cell intrinsic expression of *Chi3l1* did not appear to be required for development or expansion of the CD4 T cell compartments following *Hp* infection, we considered the possibility that T cell intrinsic expression of *Chi3l1* might instead be required for the functional or effector attributes of the T cells. To test this hypothesis, we analyzed cytokine responses by the CD4 cells from the *Hp*-infected 50:50 chimeras. We observed that the proportion of *Chi3l1* ^-/-^ CD44^hi^ CD4 cells that were competent to produce IL-4 following restimulation was significantly decreased relative to the frequency of IL-4 producing BALB/c CD4 T cells derived from the same animals (**Figures 4A, B**). Moreover, consistent with a prior publication reporting a T_H_1 differentiation bias in *Chi3l1* ^-/-^ mice (51), we observed that the proportion of CD44^hi^ *Chi3l1* ^-/-^ CD4 cells that produced IFNγ following anti-CD3 restimulation was significantly increased relative to the frequency of IFNγ-producing BALB/c CD4 T cells derived from the same animals (**Figures 4C, D**). Next, we assessed ICOS expression by the T_FH_ cells present in the *Hp*-infected 50:50 chimeras. We observed significantly decreased ICOS expression (**Figures 4E, F**) in the *Chi3l1* ^-/-^ T_FH_ cells compared to the BALB/c T_FH_ cells from the same infected animal. This was not a transient defect as it was seen even at the peak of the T_FH_ response on D14 post-infection (**Figure 4G**). These results therefore suggest that T cell intrinsic expression of Chi3l1 was important for the functional potential of the CD4 cells from *Hp*-infected mice.

One potential caveat to the 50:50 chimera experiments was that the CD45.2^+^ hematopoietic compartment would include *Chi3l1* ^-/-^ cells as well as any radiation resistant hematopoietic cells from the CD45.2^+^ WT host. The presence of these radiation resistant WT cells could potentially mask *Chi3l1* ^-/-^ cell intrinsic deficits. Although this is not a major concern for B cells, which are radiation sensitive (64), some T cell subsets are reported to be more resistant to radiation than others (reviewed in (65)). Given that we observed no differences in the frequencies or numbers of WT and *Chi3l1* ^-/-^ T_FH_ cells in the *Hp*-infected 50:50 chimeras (**Figures 3D, E**), yet we observed a reduction in ICOS levels in CD45.2^+^ T_FH_ cells from these same animals (**Figures 4E–G**), we suspected that the defect in ICOS upregulation was likely a *Chi3l1*-dependent T cell-intrinsic defect. Our *in vitro* data (**Figure 1I**) examining ICOS upregulation in activated *Chi3l1* ^-/-^ T cells also suggested a T cell intrinsic role for *Chi3l1* in regulating ICOS expression. To further confirm this conclusion, we co-cultured purified splenic CD4^+^ T cells from uninfected BALB/c CD45.1^+^ mice at a 1:1 ratio with either purified BALB/c CD45.2^+^ splenic CD4^+^ T cells or with *Chi3l1* ^-/-^ CD45.2^+^ CD4^+^ T cells in the presence of platebound anti-CD3 + anti-CD28. Again, we observed that ICOS expression by *Chi3l1* ^-/-^ CD4 T cells was significantly decreased and was not rescued in trans by the presence of the WT CD4^+^ T cells (**Figure 4H**). Taken together, these data indicate that T cell intrinsic expression of *Chi3l1* does play a role in ICOS upregulation early after TCR activation, in ICOS expression by *Hp*-induced T_FH_ cells and in IL-4 production by restimulated *Hp*-elicited CD4 T cells.

### *Chi3l1* regulates B cell responses to *Hp* infection and immunization

**Figure 5:**
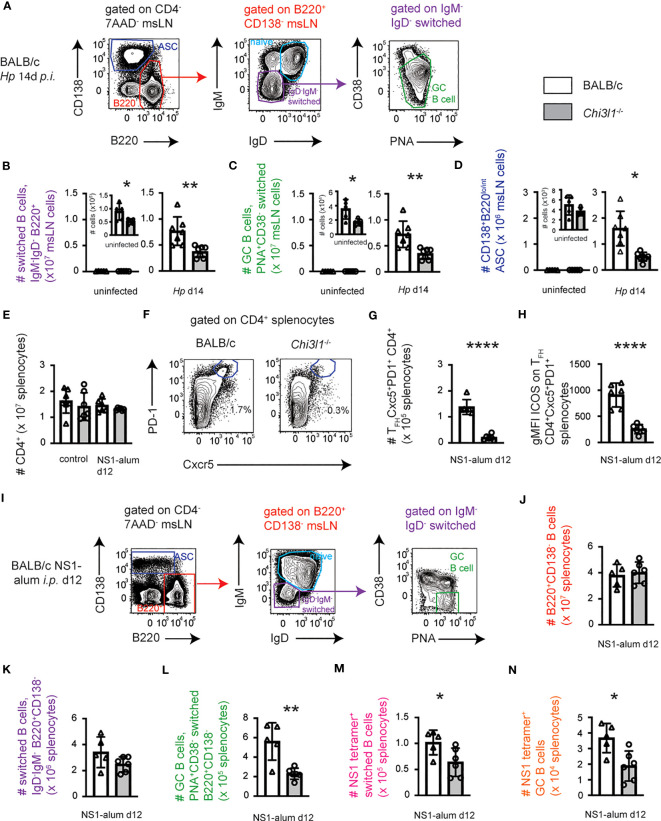
*Chi3l1* regulates B cell responses to *Hp* infection and alum-adjuvanted protein immunization. Enumeration of B cell responses in BALB/c (white bars) and *Chi3l1* ^-/-^ (grey bars) mice following infection with *Hp* **(A–D)** or vaccination with NS1 protein in alum **(E–N)**. **(A–D)** Characterization of B cell responses in msLNs of uninfected (n=5/group) and D14 *Hp*-infected (n=7/group) BALB/c and *Chi3l1* ^-/-^ mice. Flow plots **(A)** showing the gating strategy to identify B220^lo^CD138^hi^ ASCs (blue gate), total B cells (red gate), IgD^neg^IgM^neg^ isotype-switched B cells (purple gate), naïve B cells (cerulean gate) and PNA^hi^CD38^lo^ GCB cells (green gate). Numbers of isotype-switched B cells **(B)**, GCB cells **(C)**, and ASCS **(D)** in msLNs of uninfected and D14 *Hp*-infected mice are shown. Additional analyses of msLN B cell and ASC responses in *Hp*-infected WT and *Chi3l1* ^-/-^ mice (**Figure S5**) and 50:50 BM chimeras (**Figure S6**) are provided. **(E–N)** Analysis of splenic CD4 T cell **(E–H)** and B cell **(F–N)** responses in BALB/c and *Chi3l1* ^-/-^ mice (n=5-6/group) at baseline and on D12 post-immunization with influenza NS1 protein adsorbed to alum. Enumeration **(E)** of splenic CD4 T cells with representative flow plots showing the gating strategy **(F)** to identify and quantitate **(G)** splenic CXCR5^+^PD-1^+^ T_FH_ cells. ICOS expression **(H)** by T_FH_ cells reported as gMFI. Representative gating of splenic B cell subsets **(I)** and enumeration of total splenic B cells **(J)** from immunized mice. Enumeration of total splenic IgD^-^IgM^-^ isotype-switched B cells **(K)** and PNA^+^CD38^-^ GCB cells **(L)**. Numbers of NS1^+^ isotype-switched cells **(M)** and NS1^+^ GCB cells **(N)**. Representative flow plots with gating strategy to identify B cell subsets and NS1-specific B cells in spleens of control and NS1-immunized WT and *Chi3l1* ^-/-^ mice provided in **Figure S7**. Data is representative of 3 independent experiments. Data displayed as the mean ± SD of each group with individual animals depicted as circles or triangles. Statistical analysis was performed with unpaired 2-tailed student’s t-test. *p≤0.05, **p≤0.01, ****p≤0.0001.

Our data showed that Chi3L1 regulates expression of ICOS by *Hp* infection elicited T_FH_ cells and we know that ICOS is a key co-stimulatory molecule that modulates T_FH_-B cell interactions, particularly during the germinal center B cell (GCB) response (38, 39). We therefore hypothesized that *Chi3l1* ^-/-^ mice would mount impaired B cell responses to *Hp*. To test this, we analyzed B cell subsets in BALB/c and *Chi3l1* ^-/-^ msLNs before and on D14 after *Hp*-infection mice (**Figure 5A**; **Figures S5A–C**). The numbers of total msLN cells (**Figure S5D**) and B220^+^CD138^-^ B cells (**Figure S5E**) were decreased in the D14 *Hp*-infected *Chi3l1* ^-/-^ mice relative to WT mice. This reduction was not due to changes in the number of naïve msLN B cells (**Figure S5F**). Instead, antigen-experienced isotype-switched B cells (**Figure 5B**), isotype-switched GCB cells (**Figure 5C**), and antibody secreting cells (ASCs) (**Figure 5D**) were decreased in the D14 *Hp*-infected *Chi3l1* ^-/-^ msLNs. Interestingly, and unlike the phenotype of GCB cells from spleen (**Figure S7C**), msLN GCB cells in infected mice included both a CD38^lo^PNA^+^ and CD38^int^PNA^+^ population (**Figure S5G**). Both msLN GCB populations expressed similarly high levels of PNA (**Figure S5G**) and both GCB populations were present in *Hp*-infected WT and *Chi3l1* ^-/-^ msLNs (**Figure S5C**).

Since our data using 50:50 BM chimeras revealed that B cell intrinsic expression of *Chi3l1* can globally alter BM B cell development (**Figure S4**) in the setting of competition, we addressed whether B cell intrinsic expression of *Chi3l1* might be necessary for optimal GCB and ASC responses to *Hp* by analyzing D14-infected 50:50 BM chimera msLNs. In contrast to our results showing attenuated GCB and ASC responses in *Hp*-infected *Chi3l1* ^-/-^ mice (**Figures 5C, D**), we observed that *Chi3l1* ^-/-^ B cells in the 50:50 chimeras were competent to generate both ASC (**Figures S6A–C**) and GCB (**Figures S6D–K**) responses to *Hp*. This was not simply due to the presence of increased numbers of *Chi3l1* ^-/-^ B cells in the *Hp*-infected 50:50 chimeras (**Figures S6D–E**) as the frequencies of GCB cells present within the WT and *Chi3l1* ^-/-^ B cell compartments of *Hp*-infected 50:50 chimeras were elevated within the *Chi3l1* ^-/-^ B cell compartment (**Figures S6F–H**; reciprocal gating approach shown in **Figures S6I–K**). This result suggested that the reduction in the B cell responses to *Hp* in the *Chi3l1* ^-/-^ global knockout mice was not due to an intrinsic inability of the *Chi3l1* ^-/-^ B cells to enter the GCB pathway or differentiate into ASCs. Rather the defect in the *Chi3l1* ^-/-^ *Hp*-elicited B cell response in animals with a global deficiency in *Chi3l1* was likely due to the loss of *Chi3l1* by other cell types.

Next, we assessed whether the defective B cell response observed in the *Hp*-infected *Chi3l1* ^-/-^ mice was limited to the setting of helminth infection. We therefore immunized mice with recombinant protein (influenza NS1) that was adsorbed to the T_H_2-biasing adjuvant alum and used flow cytometry to measure splenic polyclonal CD4 T cell and B cell responses as well as NS1-specific B cell responses on D12 post-immunization (B cell subset flow gating strategies shown in **Figures S7A–C**). We first confirmed that numbers of splenocytes (**Figure S7D**) and splenic CD4 T cells (**Figure 5E**) did not differ in unvaccinated wildtype and *Chi3l1* ^-/-^ control mice or at D12 post-vaccination with NS1. However, the number of splenic T_FH_ cells in immunized *Chi3l1* ^-/-^ mice was significantly reduced (**Figures 5F, G**) and the remaining *Ch3l1* ^-/-^ T_FH_ cells expressed decreased levels of ICOS (**Figure 5H**). The number of splenic ASC (**Figure S7E**), percentage and number of isotype-switched B cells (**Figures S7F–G**), and number of GCB cells (**Figures S7H–I**) did not differ between WT and *Chi3l1* ^-/-^ unvaccinated control mice. Likewise, the numbers of total B cells (**Figures 5I, J**), ASC (**Figure S7E**) and switched B cells (**Figure 5K**) did not differ in vaccinated mice. In contrast, the percentages and numbers of splenic GCB cells were reduced in vaccinated *Chi3l1* ^-/-^ mice (**Figure 5L**; **Figures S7H–I**). Finally, using fluorochrome-labeled NS1 protein tetramers, we identified NS1-specific switched B cells (**Figure S7J**) and GCB cells (**Figure S7L**) in control and vaccinated WT and *Chi3l1* ^-/-^ mice. Although the frequency of NS1-specific isotype-switched (**Figure S7K**) and GCB cells (**Figure S7L**) was unchanged between the vaccinated WT and *Chi3l1* ^-/-^ mice, the numbers of NS1-specific isotype-switched B cells (**Figure 5M**) and NS1-specific GCB cells (**Figure 5N**) were significantly decreased in vaccinated *Chi3l1* ^-/-^ mice. Thus, *Chi3l1* regulates the magnitude of T_FH_ and antigen-specific B cell responses to both infection and immunization.

### *Chi3l1* regulates IgE responses

**Figure 6:**
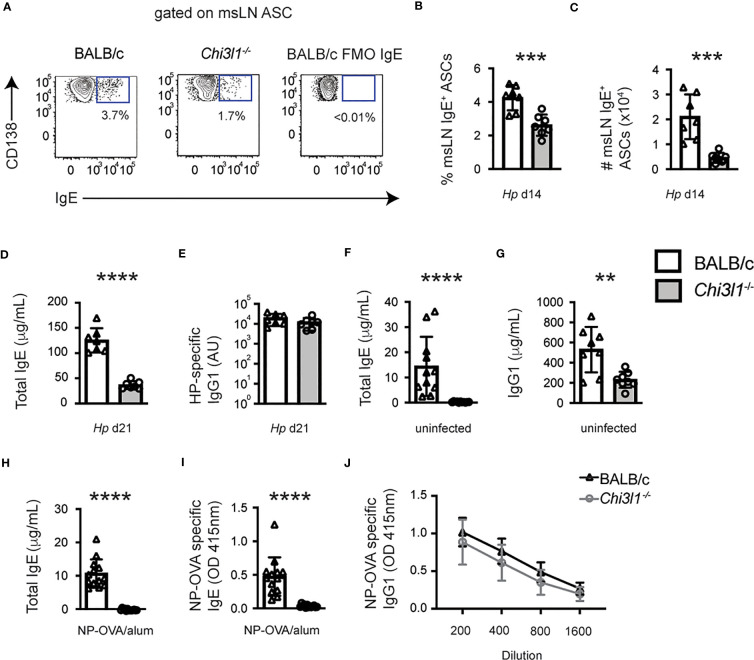
*Chi3l1* regulates IgE responses to infection and immunization. Evaluation of IgE and IgG1 antibody responses in *Hp*-infected **(A–E)**, naïve **(F–G)** and NP-OVA immunized **(H–J)** BALB/c (white bars) and *Chi3l1* ^-/-^ (grey bars) mice. **(A–C)** Quantitation of D14 IgE-expressing ASCs **(A–C)** from *Hp*-infected mice (n=7 mice/group). Representative flow plots showing msLN IgE-expressing ASCs **(A)** with the frequency **(B)** and number **(C)** of IgE-expressing ASCs. *Hp*-induced IgE^+^ ASCs in 50:50 BM chimeras are shown in **Figures S6L–N**. **(D, E)** Serum antibody levels in *Hp*-infected mice. ELISA quantitation of D21 total serum IgE **(D)** and *Hp*-specific IgG1 **(E)**. **(F, G)** Serum IgE **(F)** and IgG1 **(G)** antibody levels in uninfected mice (n=8/group). **(H–J)** Serum antibody levels in mice immunized with NP-OVA adsorbed to alum (n=13/group). Data is reported in μg/ml for total IgE **(H)** and OD values for NP-specific IgE (**I,** serum at 1:10 dilution) and NP-OVA specific IgG1 (**J**, serum diluted 1:200 to 1:1600). Data representative of 1 **(G)**, 2 **(H–J)**, 3 **(A–E)**, or 5 **(F)** independent experiments. Data displayed as the mean ± SD of each group with individual animals depicted as circles or triangles. Statistical analysis was performed with unpaired 2-tailed student’s t-test **(A–H)** or 2-way ANOVA **(J)**. P values of **(J)** is not significant (P > 0.05). **p≤0.01, ***p≤0.001, ****p≤0.0001.

Given our data and published reports showing that *Hp*-induced IgE responses depend on T_FH_ cells (35), we next analyzed IgE responses in the *Hp*-infected *Chi3l1* ^-/-^ mice. We observed a significant reduction in the frequency and number of IgE^+^ ASCs in the msLNs from *Hp*-infected *Chi3l1* ^-/-^ mice (**Figures 6A–C**) compared to the WT animals. Moreover, the impaired IgE^+^ ASC response was accompanied by a significant reduction in total IgE levels in the serum of D21 *Hp*-infected *Chi3l1* ^-/-^ mice (**Figure 6D**). In contrast, *Hp-*specific IgG1 serum antibody titers were equivalent between *Chi3l1* ^-/-^ and WT mice (**Figure 6E**). To determine whether the loss of the IgE^+^ ASCs in the *Chi3l1* ^-/-^ *Hp*-infected mice was due to a B cell intrinsic defect, we enumerated IgE-expressing ASCs within the msLN of D14 *Hp*-infected 50:50 chimeras. Consistent with the increase in *Chi3l1* ^-/-^ mature B cells in the 50:50 chimeras, we found significantly more *Chi3l1* ^-/-^ ASCs in the msLN of *Hp*-infected 50:50 chimeras (**Figure S6L**). However, the frequency of IgE ASCs within the *Chi3l1* ^-/-^ ASC compartment was equivalent to the frequency of IgE^+^ ASCs within the WT ASC compartment (**Figures S6M–N**). These data suggested that *Chi3l1* expression by non-B cells likely regulated IgE^+^ ASC responses to *Hp*.

Next, to assess whether the defective IgE response in *Chi3l1* ^-/-^ mice was restricted to *Hp* infection, we first examined serum IgE levels in non-infected animals. Although serum IgE levels were low in WT naïve mice, IgE was detected (**Figure 6F**). In contrast, IgE levels were undetectable (at least 100-fold lower) in the *Chi3l1* ^-/-^ serum (**Figure 6F**). However, total IgG1 in serum from uninfected *Chi3l1* ^-/-^ mice was only decreased ~2.5 fold relative to the WT animals (**Figure 6G**), again suggesting a more profound deficit in IgE production by *Chi3l1* ^-/-^ mice. To address whether antigen-specific IgE responses are also dependent on *Chi3l1*, we vaccinated mice with nitrophenyl haptenated ovalbumin (NP-OVA) adsorbed to alum and measured total and NP-specific IgE on D14. Again, total IgE levels were significantly lower in the vaccinated *Chi3l1* ^-/-^ mice (**Figure 6H**). Moreover, NP-specific IgE, which was easily measured in vaccinated WT mice, was undetectable in *Chi3l1* ^-/-^ serum (**Figure 6I**). In contrast, NP-specific IgG1 levels were not significant different between *Chi3l1* ^-/-^ and WT mice. (**Figure 6J**). Thus, *Chi3l1* plays a central role in IgE responses but is dispensable for IgG1 responses.

### *Chi3l1* regulates IL-4^+^ T_FH_ programming

**Figure 7:**
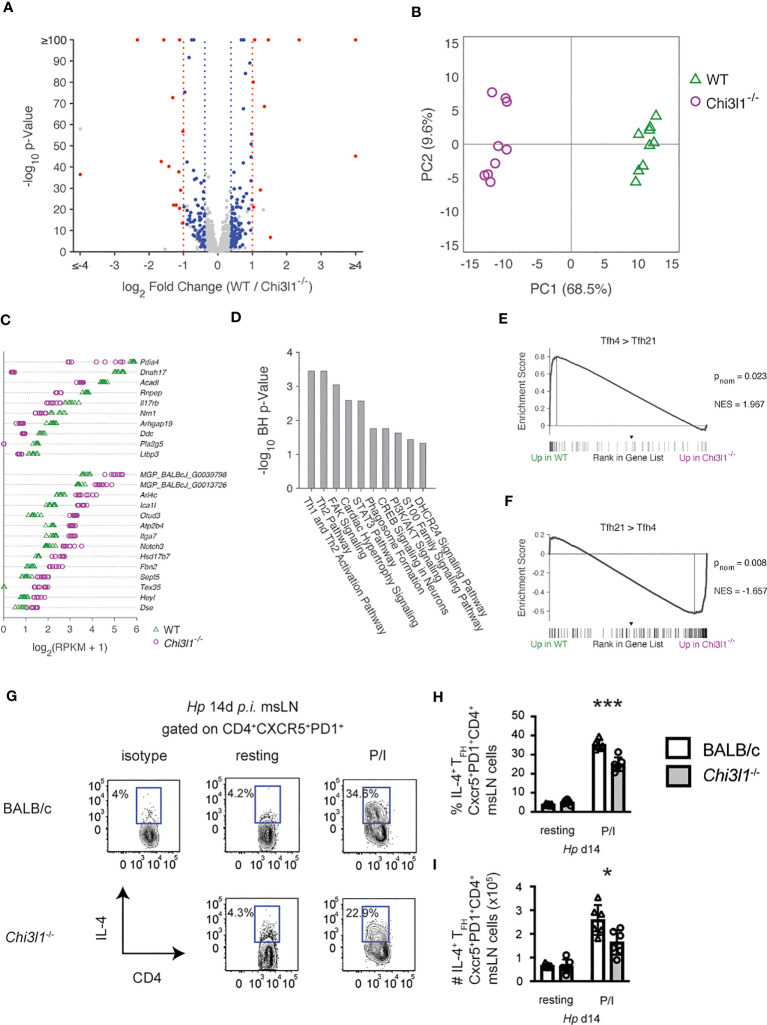
*Chi3l1* regulates T_FH4_ programming. **(A–G)** RNA-seq analysis of sorted-purified CXCR5^+^PD-1^+^ msLN T_FH_ isolated from D14 *Hp*-infected WT and *Chi3l1* ^-/-^ mice (n=9 independently pooled samples/group). See **Table S1** for gene expression levels. **(A)** Volcano plot showing 9853 expressed genes (defined as genes with at least 3 RPM in all samples of either the WT and/or *Chi3l1* ^-/-^ T_FH_). 1465 genes were expressed at significantly different levels (FDR q<0.05) between WT and *Chi3l1* ^-/-^ T_FH_ cells. Of those, genes meeting criteria of >1 RPKM in at least one group and log_2_FC of ±0.3785 (193 genes) are indicated with blue or red symbols. Red symbols indicate the 24 genes meeting a threshold of abs(log_2_FC) of ±1, blue indicate the 169 genes with abs(log2FC) between 0.3785 and 1.0. **(B)** Principal component analysis of 193 genes with > 1 mean RPKM in at least one group, ± 0.3785 log_2_FC (1.3-fold) and FDR q<0.05. **(C)** Absolute expression values of 24 genes with > 1 mean RPKM in at least one group, ± 1.0 log_2_FC (2-fold) and FDR q<0.05. **(D)** The gene list containing 193 genes > 1 RPKM in at least one group meeting an FDR q< 0.05 and ± 0.3785 log_2_FC cutoff were imported into Ingenuity Pathway Analysis (IPA, QIAGEN Digital Insights) to identify significant predicted signaling pathways. 182 genes were analyzed by IPA. Pathways with a Benjamini-Hochberg **(B–H)** corrected overlap p< 0.05 are shown. **(E, F)** Gene set enrichment analysis (GSEA) (50) using the ranked gene list of WT and *Chi3l1* ^-/-^ T_FH_ cells and DEG identified as up in IL4^+^IL-21^neg^ T_FH_ cells (T_FH4_, panel **E**) or up in IL-4^neg^IL-21^+^ T_FH_ cells (T_FH21_, panel **F**) isolated from *Nippostrongylus brasiliensis*-infected mice (40). p_nom_ and NES are provided. **(G–I)** Cytokine production by D14 *Hp*-infected WT and *Chi3l1* ^-/-^ T_FH_ cells (N=5 mice/group) following *in vitro* PMA+ionomycin restimulation. Flow plots **(G)** measuring IL-4 production and the percentage **(H)** and number **(I)** of T_FH_4 cells in each culture. Data representative of 3 independent experiments **(G–I)** or one experiment **(A–F)** with 9 independent RNA-seq samples/group. Data in **(G–I)** displayed as the mean ± SD of each group with individual animals depicted as circles or triangles. Statistical tests for RNA-seq data described in the legends. Statistical analysis for **(G–I)** was performed with unpaired 2-tailed student’s t-test. *p≤0.05, ***p≤0.001.

IL-4 producing T_FH_ cells are required for the polyclonal IgE response to *Hp* (35). Since B cell intrinsic expression of *Chi3l1* was not required for the IgE ASC response to *Hp* (**Figures S6L–N**) and T_FH_ cells were decreased in the *Hp*-infected *Chi3l1* ^-/-^ mice, we suspected that the large reduction in IgE responses seen in these mice might reflect additional functional impairments in the *Chi3l1* ^-/-^ T_FH_ cells. To assess this in an unbiased fashion, we compared the transcriptome of T_FH_ cells from msLNs of WT and *Chi3l1* ^-/-^ mice on D14 post *Hp* infection. We identified 9853 expressed genes and 1465 differentially expressed genes (DEG) between the two T_FH_ populations that met an FDR q<0.05 cutoff (**Table S1**). 252 DEG exhibited at least a ±0.3785 log_2_ fold-change (log_2_FC) (**Figure 7A**), with 193 of these genes expressed at >1 mean RPKM in either BALB/c or *Chi3l1* ^-/-^ T_FH_. Principal component analysis based on these 193 DEGs shows clear sample group separation (**Figure 7B**). Since the differences in expression levels between most of the genes expressed by WT and *Chi3l1* ^-/-^ T_FH_ cells was relatively modest, with only 24 of the DEG with RPKM > 1 meeting a ± 1 log_2_FC threshold (**Figures 7A, C**), we reasoned that we might observe smaller changes in gene expression across many genes within specific pathways. To test this, we performed Gene Set Enrichment Analysis (GSEA) (50) comparing the rank-ordered WT and *Chi3l1* ^-/-^ T_FH_ gene set to the 5219 C7 Immunologic Signature Gene Sets from MSigDB. None of the C7 gene sets were preferentially enriched (FDR q<0.05) in the WT or *Chi3l1* ^-/-^ T_FH_ transcriptomes, including the 54 C7 gene sets derived from T_FH_ cells (*data not shown*). This result, which was consistent with normal expression of the T_FH_ lineage master regulator Bcl-6 by *Chi3l1* ^-/-^ T_FH_ cells (**Figures 1D, E**), indicated that *Chi3l1* is not required for establishment of the core T_FH_ transcriptional program (66). We next performed Ingenuity Pathway Analysis (IPA) with the 193 genes meeting an FDR q<0.05 and ± 0.3785 log_2_FC threshold to assess whether changes in expression of suites of genes might reflect alterations in *Chi3l1* ^-/-^ T_FH_ cell function or signaling. IPA-defined pathways that were predicted to be most significantly different (B-H p < 0.05) between the WT and *Chi3l1* ^-/-^ T_FH_ cells included T_H_2 and T_H_1/T_H_2 activation (**Figure 7D**).

IL-4 produced by T_FH_ cells is required for B cell class switching to IgE following *Hp* infection (35, 67) and even modest reductions in the amount of IL-4 present can prevent B cell switching to IgE while having no impact on the IgG1 response (68). Prior studies examining the T_FH_ response after infection with the nematode *Nb* showed that T_FH_ cells change over time following infection and proceed from producing IL-21 alone (T_FH21_ cells) to producing primarily IL-4 (T_FH4_ cells) (40). This change in the T_FH_ cytokine profile from IL-21 to IL-4 is associated with migration of T_FH_ cells into the B cell follicle (69), formation of ICOS-regulated T_FH_/B cell conjugates that support T_FH_ cell survival and expansion (70), and acquisition of a transcriptionally distinct T_FH4_ program (40). Given our data, we hypothesized that Chi3l1 might regulate acquisition of the T_FH4_ transcriptional program. To assess this, we performed GSEA using published lists of genes that are differentially expressed in T_FH4_ and T_FH21_ cells from *Nb-*infected mice (40). We found that DEG that are normally upregulated in *Nb* T_FH4_ cells compared to *Nb* T_FH21_ cells (40) were significantly enriched in *Hp* WT T_FH_ cells compared to *Hp Chi3l1* ^-/-^ T_FH_ cells (**Figure 7E**). In contrast, we observed significant enrichment in the transcriptome of the *Hp*-induced *Chi3l1* ^-/-^ T_FH_ cells for genes that are increased in *Nb* T_FH21_ cells relative to *Nb* T_FH4_ cells (**Figure 7F**). Finally, to address whether loss of *Chi3l1* also altered IL-4 production specifically by T_FH_ cells, we measured IL-4 in PMA+ionomycin- restimulated CXCR5^+^PD-1^hi^ T_FH_ cells from D14 *Hp*-infected WT and *Chi3l1* ^-/-^ mice. Consistent with the GSEA data, we observed a significant decrease in frequency and number (**Figures 7G–I**) of *Chi3l1* ^-/-^ T_FH4_ cells relative to WT T_FH4_ cells. Together, the data support the conclusion that *Chi3l1* contributes to the development or maintenance of the ICOS-expressing T_FH4_ subset that facilitates B cell IgE responses.

## Discussion

Studies of human allergic diseases reveal that expression of YKL-40 protein and *CHI3L1* transcripts are increased in the lungs and serum of some asthma cohorts (13, 14). Increased expression of YKL-40/*CHI3L1* is positively associated with pathogenesis in allergic rhinitis (19, 20), atopic dermatitis (21–23) and food allergy (24) and *CHI3L1* SNPs confer risk of asthma development and airway remodeling (14–17) as well as increased serum IgE and atopy (18). Similarly, experiments using *Chi3l1* ^-/-^ mice show that Chi3l1 regulates type 2 cytokines and IgE in models of asthma, atopic dermatitis and food allergy (8, 23–26). Thus, both mouse and human data support a role for Chi3l1 in promoting atopic disease. Here, we extend these prior studies to show that *Chi3l1* also facilitates T_H_2 responses to the pathogen *Hp*. In addition, we identify Chi3l1 as a key regulator of T_FH_ responses following both *Hp* infection and alum-adjuvanted vaccination and demonstrate that Chi3l1, while not required for T_FH_ development, plays critical roles in T_FH_ expansion, ICOS expression by T_FH_ cells, IL-4 production by T_FH_ cells and T_FH_-dependent IgE production.

Lower organisms utilize chitinases and chitinase-like proteins (CLPs) in innate host defense against chitin-containing pathogens. In *Drosophila*, the transport of secreted CLP homologues *via* the hemolymph as well as expression of CLP by hemocytes, the evolutionary precursors of mammalian leukocytes, mediate host defense (71). Following infection, the CLP homologue is transcriptionally upregulated (72), resulting in increased hemolymph levels (73, 74). In parallel, mammalian CLPs are theorized to function as soluble mediators that can be expressed and secreted by activated hematopoietic and damaged mesenchymal cells (75, 76). Consistent with this, our data suggested that Chi3l1 can function as a cytokine-like paracrine regulator in some settings. However, we also observed cell-intrinsic roles for Chi3l1 in CD4 T cell function and progenitor B cell development, suggesting that Chi3l1 might signal in an autocrine manner. Consistent with prior reports (51), we observed no difference in the size of the mature CD4 T cell in mice globally deficient in *Chi3l1*. Likewise, the number of WT and *Chi3l1* ^-/-^ mature msLN CD4 T cells and splenic CD4 T cells, CD8 T cells and myeloid cells were similar in uninfected 50WT:50*Chi3l1* ^-/-^ chimeras, indicating that Chi3l1 expression by T and myeloid cells is not required for their development. In contrast, when *Chi3l1* ^-/-^ B lineage BM precursors were placed in direct competition with WT B lineage precursors, we observed that the mature peripheral naïve B cell compartment was dominated by *Chi3l1* ^-/-^ B cells. This effect was evident as early as the pre-B cell stage in the BM, suggesting that Chi3l1 can play a B cell intrinsic role as a negative regulator of B cell development and that this intrinsic property becomes evident when WT and *Chi3l1* ^-/-^ B cell precursors are forced to compete in the competitive BM chimera setting.

Following *Hp* infection, we observed significant decreases in size of the CD4 T cell, T_FH_ cell, and GCB cell responses in the *Chi3l1* global knockouts. These defects appeared to be rescued in the LNs of *Hp*-infected 50WT:50*Chi3l1* ^-/-^ BM chimeras, suggesting that intrinsic expression of *Chi3l1* by T cells is not important for the development or expansion of T_FH_ cells. However, given the way that we generated the 50WT:50*Chi3l1* ^-/-^ BM chimeras, we can’t absolutely exclude a T cell-intrinsic role for *Chi3l1* in T_FH_ development. What we do know is that *Chi3l1* expression by hematopoietic cells was both necessary and sufficient to elicit CD4 T cells responses to *Hp*. These results, which are complimentary to a study showing that hematopoietic lineage expression of *Chi3l1* was sufficient to rescue airway inflammation in *Chi3l1* ^-/-^ mice sensitized to *Aspergillus fumigatus* conidia (25), suggest that Chi3l1-producing hematopoietic cells support expansion of *Hp*-induced T_FH_ and CD4 T cells.

Although Chi3l1 expression by T cells does not control the size of the CD4 T cell and T_FH_ response to *Hp*, our data show that multiple attributes of T_FH_ cells are dependent on T cell intrinsic expression of Chi3l1. Indeed, despite equivalent numbers of WT and *Chi3l1* ^-/-^ CD4 T cells and T_FH_ cells in *Hp*-infected 50WT:50*Chi3l1* ^-/-^ BM chimeras, the frequency of CD4 T cells competent to produce IL-4 within the *Chi3l1* ^-/-^ CD4 compartment was decreased by almost 50% relative to the WT CD4 T cell compartment. These data fit well with prior studies showing that *Chi3l1* ^-/-^ CD4 T cells primed *in vitro* with antibodies to CD3 and CD28 in the presence of T_H_2-polarizing conditions are impaired in IL-4 production (51). Moreover, both our *in vitro* and *in vivo* experiments reveal that ICOS upregulation by naïve T cells and T_FH_ cells requires T cell-specific expression of *Chi3l1*. Given that both ICOS and Chi3l1 are upregulated by WT CD4 T cells following TCR and CD28 engagement (51, 77–79) and *Chi3l1* ^-/-^ CD4 T cells are impaired in ICOS expression after *in vitro* stimulation with plate-bound antibodies to CD3 and CD28, we think it likely that Chi3l1 enhances ICOS expression by modulating signaling downstream of TCR and/or CD28 engagement. *Chi3l1* ^-/-^ CD4 T cells are reported to be hyperresponsive to anti-CD3+CD28 ligation *in vitro* (51), suggesting that Chi3l1 functions as a negative regulator. This is intriguing given a recent report showing that low tonic TCR signaling is associated with increased ICOS expression by pre-T_FH_, enhanced T_FH_ development and more robust GCB cell responses (80).

While T_FH_ responses to *Hp* infection and vaccination are clearly impaired in *Chi3l1* ^-/-^ mice, we do not think that this is due to a requirement for *Chi3l1* during commitment to the T_FH_ lineage as the remaining msLN *Chi3l1* ^-/-^ T_FH_ cells express normal levels of the master T_FH_ regulator Bcl-6 (66) and the core T_FH_ transcriptional program (66) appears intact. Instead, we find that fewer *Chi3l1* ^-/-^ T_FH_ cells progress to becoming functional IL-4 producing T_FH_ cells (T_FH4_ cells), which are normally induced following *Hp* infection (35). This is due, at least in part, to an inability of these cells to acquire the mature T_FH4_ transcriptional program (40). Instead, *Chi3l1* ^-/-^ T_FH_ cells appear to be transcriptionally enriched in genes expressed by IL-21 and the IL-21 plus IL-4 producing T_FH_ cells (40). Prior studies examining the T_FH_ response after infection with the nematode *Nippostrongylus brasiliensis* showed that T_FH_ cells change over time following infection and proceed from producing IL-21 alone to producing primarily IL-4 (40). This change in the T_FH_ cytokine profile from IL-21 to IL-4 coincides with the migration of T_FH_ cells into the B cell follicle (69) and the formation of T_FH_/B cell conjugates (70). Our data therefore suggest that *Chi3l1* is required for the full maturation of the T_FH_ response from T_FH21_ to B cell-engaged T_FH4_ cells.

Although our data do not specify the mechanism by which Chi3l1 controls the expansion and maturation of the T_FH4_ response to *Hp* infection, we suspect that it is related to decreased expression of ICOS by *Chi3l1* ^-/-^ CD4 T_FH_ cells. It is well appreciated that ICOS/ICOSL interactions between B cells and T_FH_ cells is required for the maintenance of the T_FH_ population in the B cell follicle and GC (81) and that blockade of ICOS/ICOSL in established allergic responses is sufficient to ablate ongoing T_FH_ and GC responses (82). Moreover, data from parasite infection models indicate that IL-4 production by T_FH_ cells not only requires B cells (34) but is dependent on ICOS/ICOSL interactions within the B cell follicle (70). Thus, we think that Chi3l1, which is upregulated in CD4 T cells upon TCR stimulation (51), induces ICOS expression in a cell intrinsic manner. ICOS-expressing T_FH_ cells can then productively engage with ICOSL-expressing B cells, which in turn provide T_FH_ cells with appropriate co-stimulatory signals to support their expansion, maintenance and acquisition of the T_FH4_ transcriptional program within the B cell follicle (70, 81–83). Given the known role for ICOS-ICOSL interactions in supporting T_FH_-dependent B cell responses (81), we argue that the decrease in antigen-specific B cell responses in vaccinated *Chi3l1* ^-/-^ mice is also due, in large part, to the poor expansion of the T_FH_ compartment and the reduction in ICOS expression by the remaining *Chi3l1* ^-/-^ T_FH_ cells.

One of the most striking defects in naïve, vaccinated and *Hp*-infected *Chi3l1* ^-/-^ mice is the near total ablation of secreted IgE and IgE-expressing ASCs. The data from the *Hp*-infected 50WT:50*Chi3l1* ^-/-^ mice indicate that this is not due to the loss of Chi3l1 expression by B lineage cells. We propose the loss of the IgE response in vaccinated and infected *Chi3l1* ^-/-^ mice is most likely due to the impaired IL-4 production by the T_FH_ as it was reported that IL-4 produced specifically by T_FH_ cells is required for B cell class switching to IgE following *Hp* infection (35, 67). We think that poor conversion of T_FH21_ cells to the T_FH4_ subset may also explain why *Hp*-infected *Chi3l1* ^-/-^ mice make relatively normal IgG1 responses. It is reported that IL-21 promotes switching to IgG1 and actively inhibits switching to IgE (84) and that even modest reductions in the amount of IL-4 produced can prevent B cell switching to IgE while having no impact on the IgG1 response (68). Although we think that the loss of the IgE response in *Chi3l1* ^-/-^ mice is due to the impaired T_FH4_ response, ICOS/ICOSL interactions also contribute to the development of both IL-4 producing T cells and IgE responses following allergen sensitization and challenge (83) and blockade of ICOS/ICOSL in established allergic responses is sufficient to impair IgE production (82). Therefore, it is possible that Chi3l1 tunes ICOS expression levels, which favors IL-4 production by T_FH_ cells and supports isotype switching of the ICOSL-expressing B cells to IgE.

In summary, our data reveal new roles for Chi3l1 in controlling upregulation of ICOS by T_FH_ cells, expansion of the T_FH_ compartment, acquisition of the T_FH4_ transcriptional program, establishment of T_FH_-dependent B cell responses and production of secreted IgE. We show that these effects are due to paracrine hematopoietic cell derived Chi3l1 as well as T cell intrinsic Chi3l1. These data provide avenues for future research into the mechanisms by which Chi3l1 exerts its various functions in allergic responses and suggest that directed approaches that block Chi3l1 activity or function specifically within the T cell compartment may attenuate both T_H_2 responses and T_FH_-driven IgE responses in pathologic settings such as atopy and allergic airway disease.

## Data availability statement

The datasets presented in this study can be found in online repositories. The names of the repository/repositories and accession number(s) can be found below: GSE203113 (GEO); https://www.ncbi.nlm.nih.gov/geo/query/acc.cgi?acc=GSE203113.

## Ethics statement

The animal study was reviewed and approved by University of Alabama Birmingham Institutional Animal Care and Use Committee (IACUC).

## Author contributions

Conceptualization, MC and FL, Methodology, MC and JB, Investigation and Analysis, MC, NB, AR, CDS, JB, and BM, Writing – Original Draft: MC and FL, Writing – Review and Editing: MC, FL, CDS, and AR, Funding Acquisition: FL, TR, and MC, Resources, CDS, BL, TR, and AR, Supervision, MC and FL. All authors contributed to the article and approved the submitted version.
