# Supplementary figures and images for: Chitinase-3-like 1 regulates TH2 cells, TFH cells and IgE responses to helminth infection
Source: Front Immunol. 2023 Jul 27;14:1158493. doi: 10.3389/fimmu.2023.1158493 (PMC10415220; doi:10.3389/fimmu.2023.1158493)

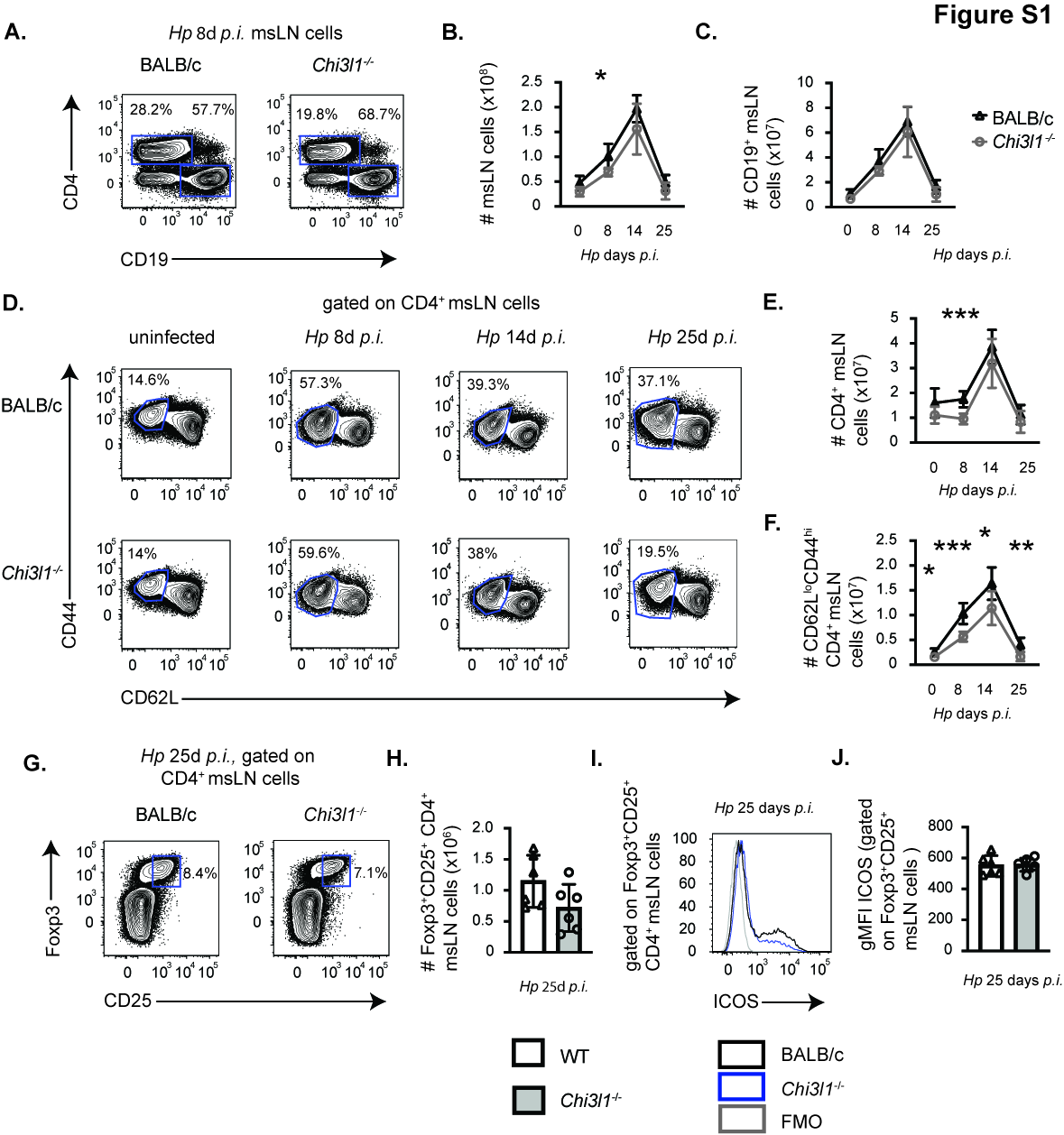

Supplement: Supplementary file 1 [file Image_1.tif]

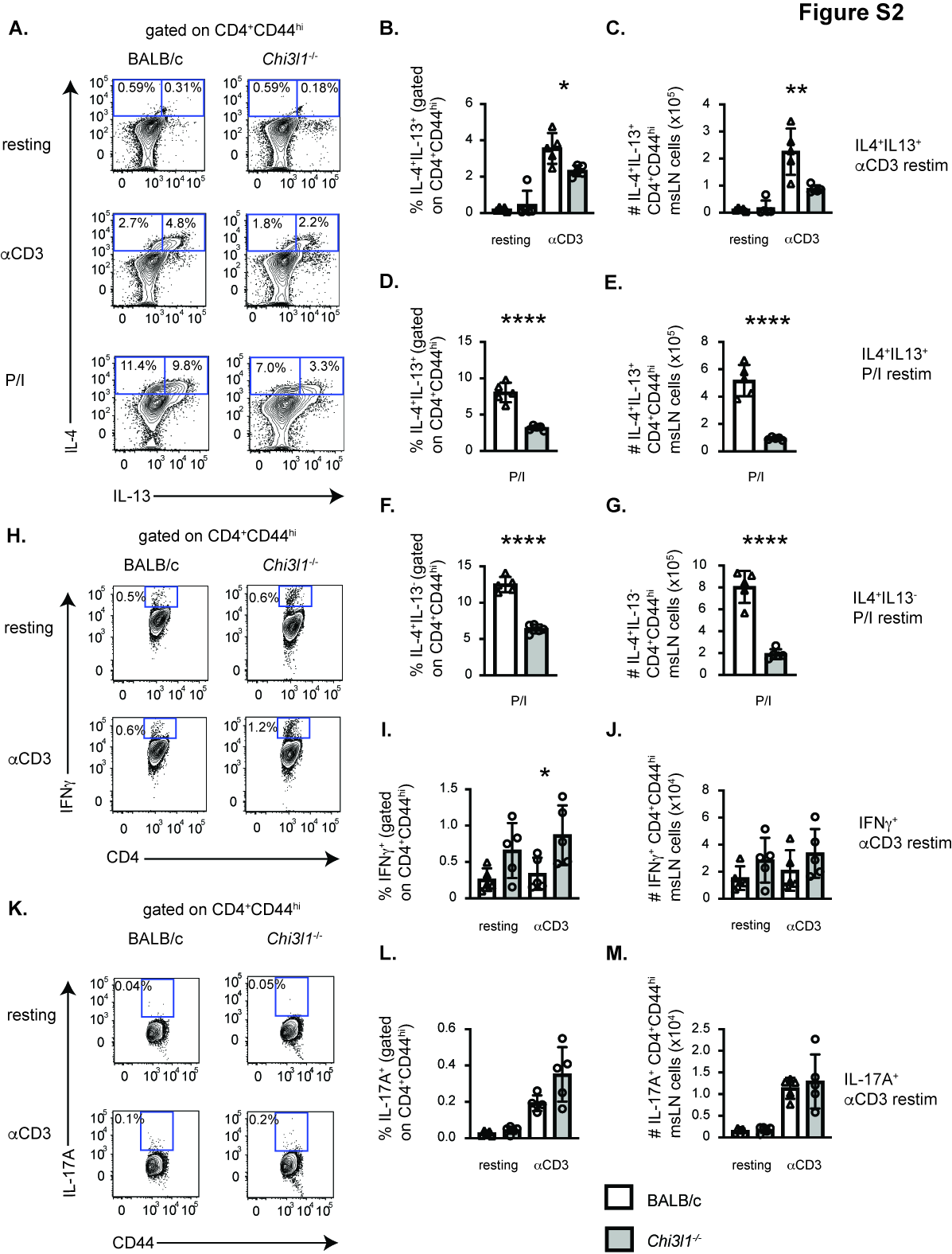

Supplement: Supplementary file 2 [file Image_2.tif]

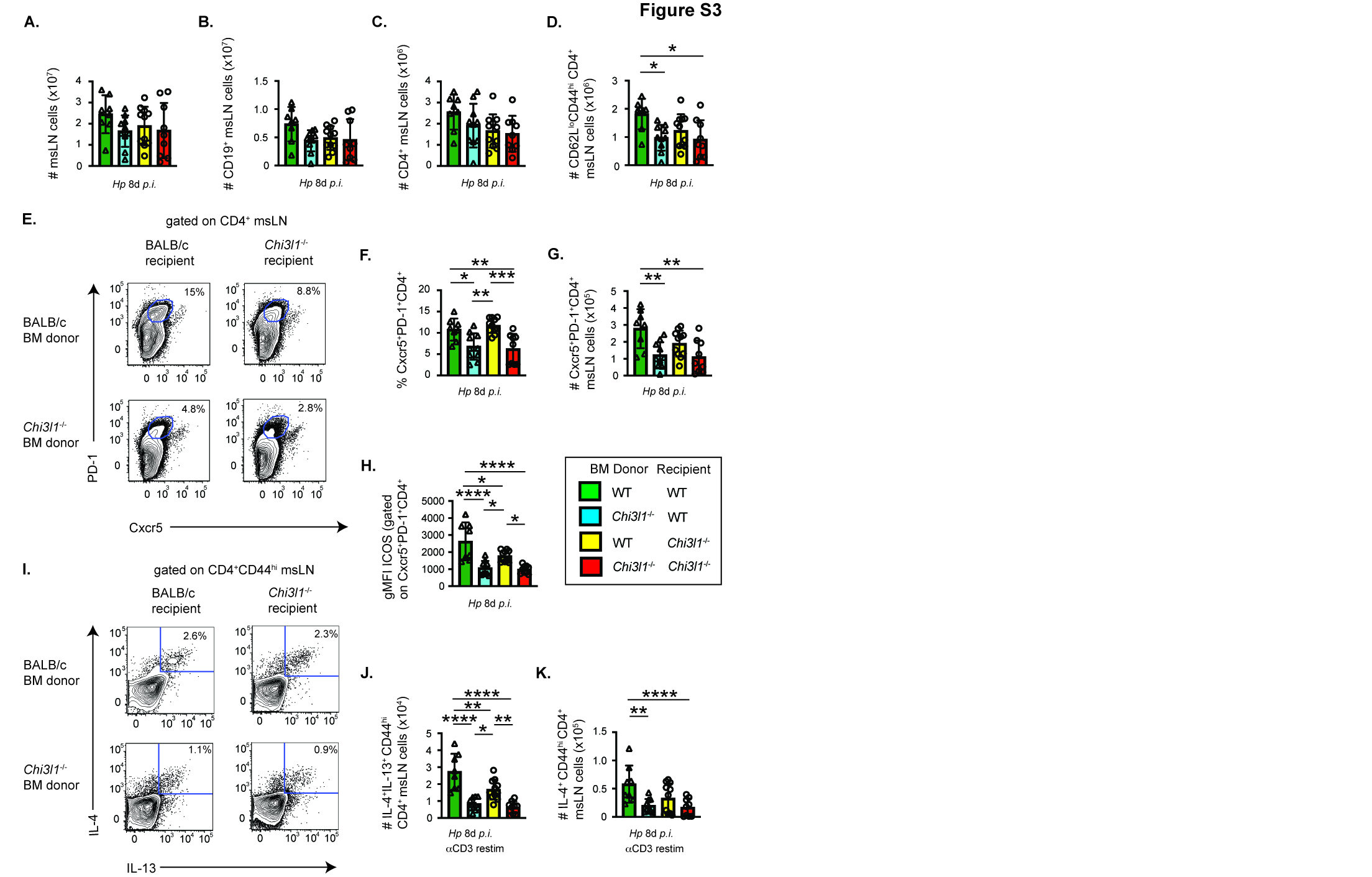

Supplement: Supplementary file 3 [file Image_3.tif]

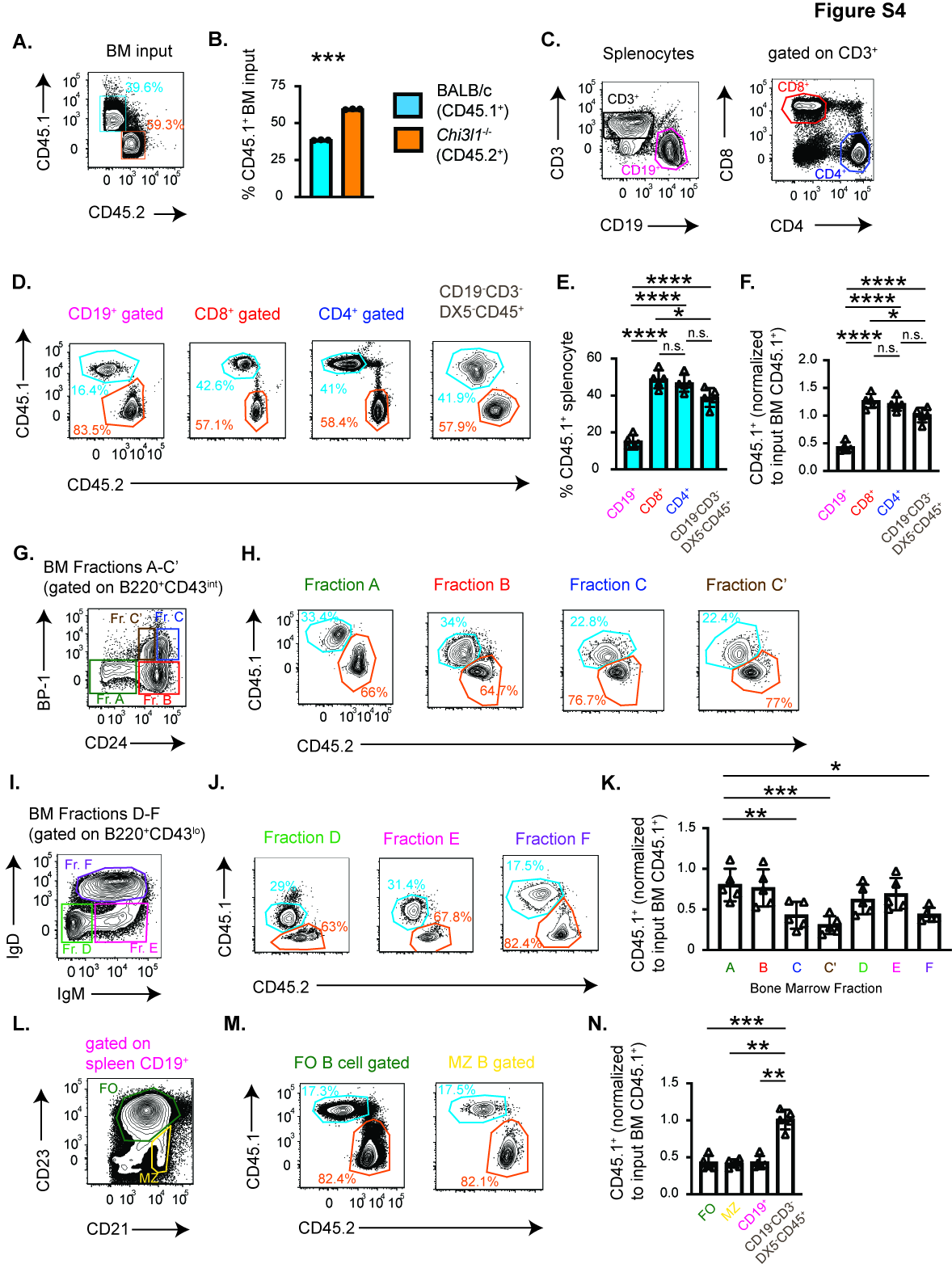

Supplement: Supplementary file 4 [file Image_4.tif]

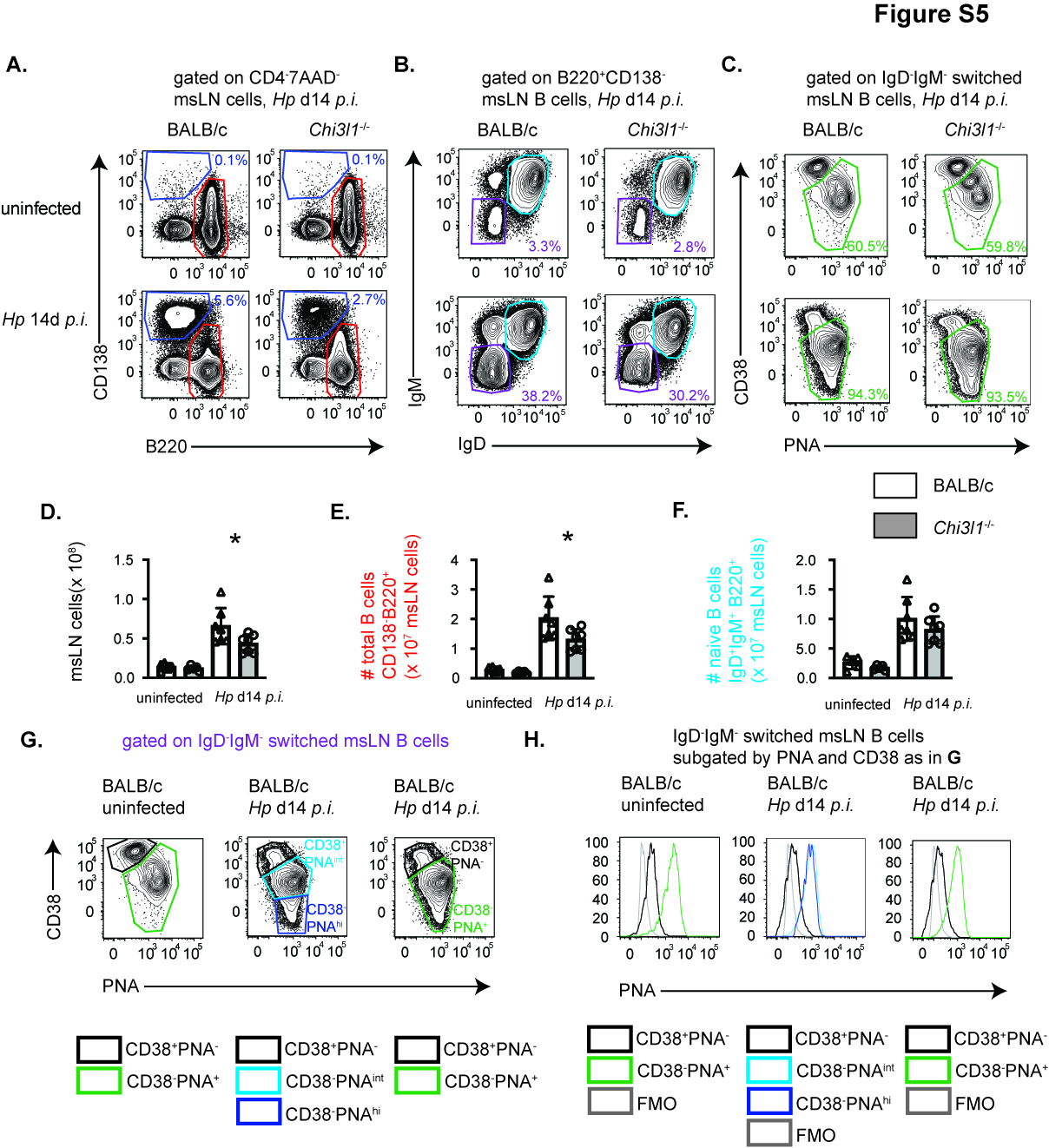

Supplement: Supplementary file 5 [file Image_5.tif]

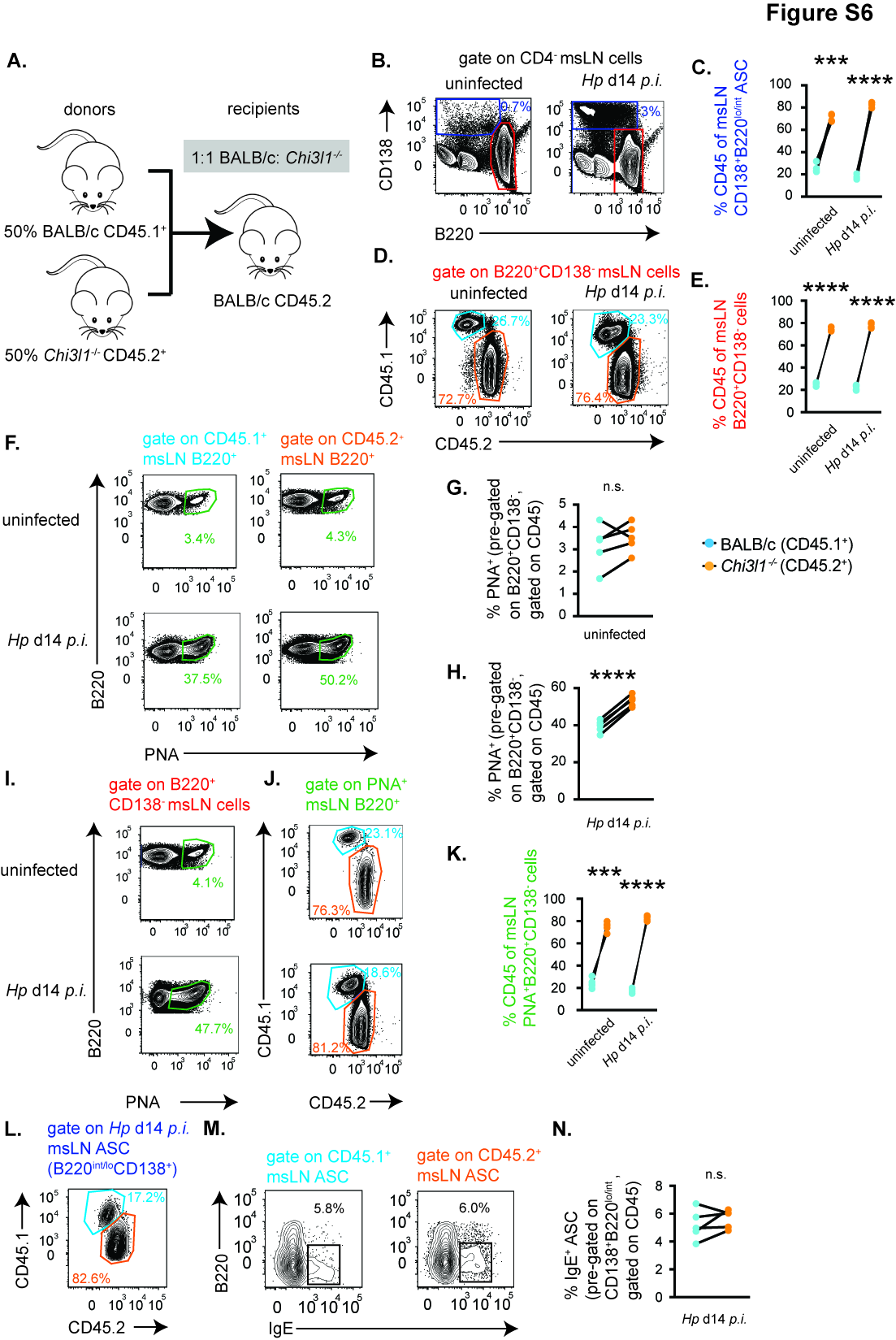

Supplement: Supplementary file 6 [file Image_6.tif]

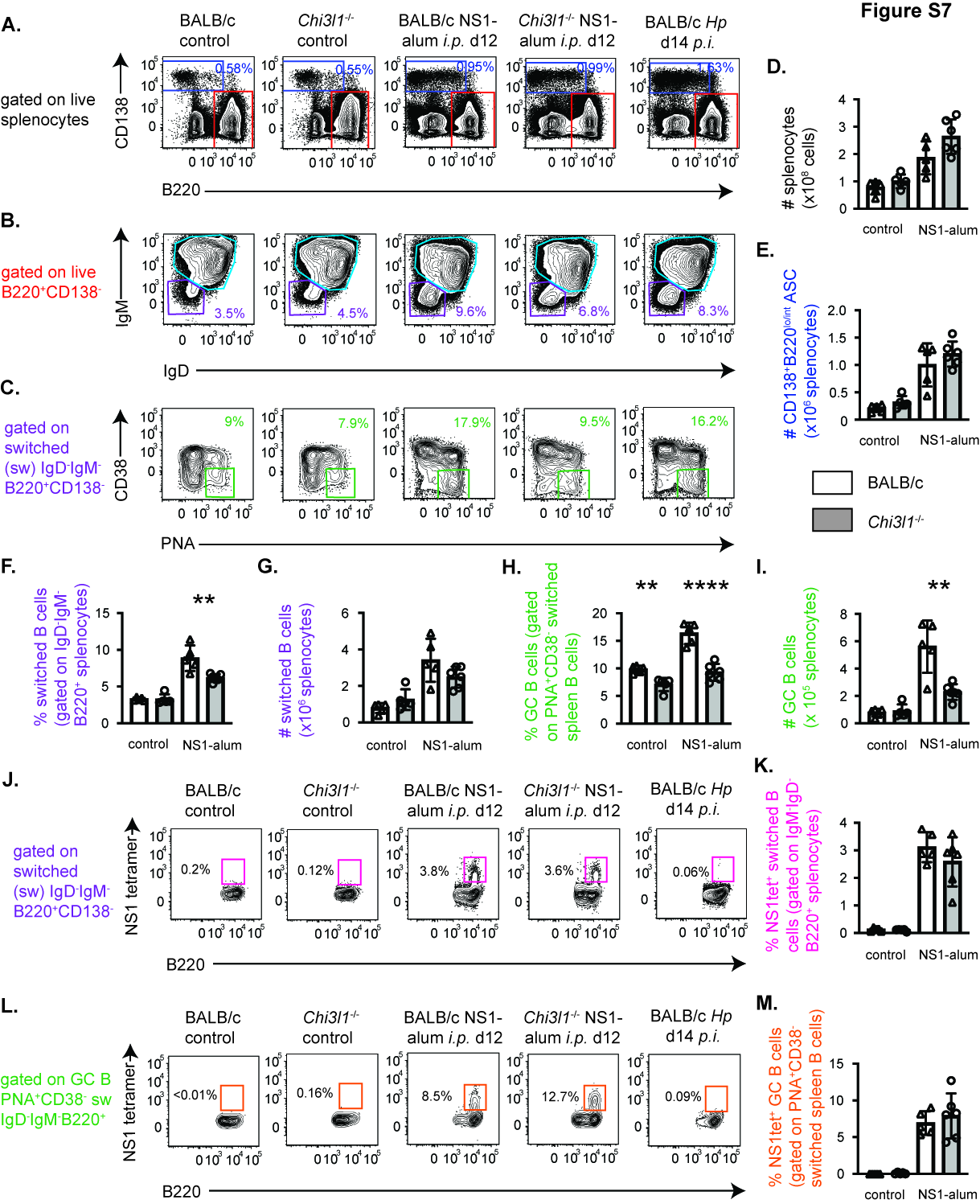

Supplement: Supplementary file 7 [file Image_7.tif]
