# Supplementary material for: Chitinase-3-like 1 regulates TH2 cells, TFH cells and IgE responses to helminth infection
Source: Front Immunol. 2023 Jul 27;14:1158493. doi: 10.3389/fimmu.2023.1158493 (PMC10415220; doi:10.3389/fimmu.2023.1158493)
Supplement: Supplementary file 9 [file DataSheet_1.pdf]

Supplementary Material

Chitinase-3-like 1 regulates T_H2 cells, T_{FH} cells and IgE responses to helminth infection

Miranda L. Curtiss[1*], Alexander F. Rosenberg[2,3], Christopher D. Scharer[4], Betty Mousseau[2], Natalia A. Ballesteros Benavides[1,2], John E. Bradley[5], Beatriz León[2], Chad Steele[6], Troy D. Randall[5], Frances E. Lund[2]

*** Correspondence:** Corresponding Author: mlcurtiss@uabmc.edu

Supplementary Table 1

Table S1 Supporting Figure 8. RNA-seq methods and analysis of msLN T_{FH} cells isolated from D14 *Hp*-infected WT and *Chi3l1*$^{-/-}$ mice. RNA-seq libraries were prepared from sort-purified msLN T_{FH} cells derived from D14 *Hp*-infected WT and *Chi3l1*$^{-/-}$ mice (samples derived from 3 independent experiments with 2-3 mice/group/experiment). FDR, p value and log_2FC values are provided for all expressed genes, which are defined as genes with at least 3 reads per million in all samples of at least one group.

frontiers

Supplementary Material

Chitinase-3-like 1 regulates T_H2 cells, T_{FH} cells and IgE responses to helminth infection

Miranda L. Curtiss[1*], Alexander F. Rosenberg[2,3], Christopher D. Scharer[4], Betty Mousseau[2], Natalia A. Ballesteros Benavides[1,2], John E. Bradley[5], Beatriz León[2], Chad Steele[6], Troy D. Randall[5], Frances E. Lund[2]

*** Correspondence:** Corresponding Author: mlcurtiss@uabmc.edu

Supplementary Figure 1

Supplemental Figure S1 showing supporting information for Figure 1. CD62L^{lo}CD44^{hi} activated CD4 cells but not Foxp3^{+}CD25^{+} CD4 T_{REG} are decreased in *Chi3l1$^{-/-}$* mice following *Hp* infection. (**A-F**) Enumeration and characterization of CD4 T cell subsets in msLNs of uninfected (n=5/group) and D8 *Hp*-infected (n=5/group/timepoint) BALB/c (white bars) and *Chi3l1$^{-/-}$* (grey bars) mice. Kinetic analysis of total msLN cells and lymphocytes between D0 (uninfected) and D25 gated as shown in (**A**). Data reported as number of total msLN cells (**B**), CD19^{+} B cells (**C**), and CD4^{+} T cells (**E**). Representative gating (**D**) for kinetic analysis of activated CD62L^{lo}CD44^{hi} CD4 cells (**F**) at each timepoint. (**G-J**) Enumeration and characterization of CD4^{+}Foxp3^{+}CD25^{+} regulatory (T_{REG}) T cells in msLNs of D25 *Hp*-infected BALB/c and *Chi3l1$^{-/-}$* mice. Quantification of total D25 msLN CD25^{+}Foxp3^{+}CD4^{+} T_{REG} cells (**H**), gated as shown in (**G**), and (**J**) the gMFI of ICOS expression by the D25 CD25^{+}Foxp3^{+} cells. Histograms (**I**) showing ICOS staining by CD25^{+}Foxp3^{+} T_{REG} cells. FMO control is shown in grey. Data representative of ≥ 3 independent experiments. Data displayed as the mean±SD of each group. Statistical analysis was performed with unpaired 2-tailed student's t-test at each timepoint. *$p \leq 0.05$, **$p \leq 0.01$, ***$p \leq 0.001$, ****$p \leq 0.0001$.

Supplementary Material

Chitinase-3-like 1 regulates T_H2 cells, T_{FH} cells and IgE responses to helminth infection

Miranda L. Curtiss[1*], Alexander F. Rosenberg[2,3], Christopher D. Scharer[4], Betty Mousseau[2], Natalia A. Ballesteros Benavides[1,2], John E. Bradley[5], Beatriz León[2], Chad Steele[6], Troy D. Randall[5], Frances E. Lund[2]

*** Correspondence:** Corresponding Author: mlcurtiss@uabmc.edu

Supplementary Figure Legends

Supplemental Figure S2 showing supporting information for Figure 1. CD4 effector type 2 cytokine responses are impaired in *Hp*-infected *Chi3l1*$^{-/-}$ mice. Cytokine production by CD4 cells from the msLN of D8 *Hp*-infected (n=5/group) BALB/c (white bars) and *Chi3l1*$^{-/-}$ (grey bars) mice cultured for 4 hours with Brefeldin A (BFA). Intracellular IL-4, IL-13, IFNγ and IL-17A were measured in CD44^{hi} gated CD4 T cells that were rested or restimulated for 4 hours with plate-bound anti-CD3 (**A-C, H-M**) or PMA and ionomycin (**D-G**). (**A-C**) IL-4 and IL-13 production by resting and anti-CD3 stimulated msLN CD44^{hi} CD4 cells (**A**) with the percentage and number of IL-4$^+$IL-13$^+$ (**B-C**) producers. Percentage and number of IL-4$^+$IL-13$^+$ cells (**D-E**) and IL-4$^+$IL-13^{neg} cells (**F-G**) restimulated with PMA and ionomycin. (**H-J**) IFNγ production by resting and anti-CD3 stimulated msLN CD44^{hi} CD4 cells (**H**) with the percentage and number of IFNγ$^+$ (**I-J**) producers. (**K-M**) IL-17A production by resting and anti-CD3 stimulated msLN CD44^{hi} CD4 cells (**K**) with the percentage and number of IL-17A$^+$ (**L-M**) producers. Data representative of ≥ 3 independent experiments and displayed as the mean±SD of each group with individual animals depicted as circles or triangles. Statistical analysis was performed with unpaired 2-tailed student's t-test. *p≤0.05, **p≤0.01, ***p≤0.001, ****p≤0.0001.

Supplementary Material

Chitinase-3-like 1 regulates T_H2 cells, T_{FH} cells and IgE responses to helminth infection

Miranda L. Curtiss[1*], Alexander F. Rosenberg[2,3], Christopher D. Scharer[4], Betty Mousseau[2], Natalia A. Ballesteros Benavides[1,2], John E. Bradley[5], Beatriz León[2], Chad Steele[6], Troy D. Randall[5], Frances E. Lund[2]

*** Correspondence:** Corresponding Author: mlcurtiss@uabmc.edu

Supplementary Figure 3

Supplemental Figure 3 supporting Figure 2. *Chi3l1* expressing hematopoietic cells are sufficient for T_{FH} and T_H2 responses to *Hp*. BM chimeric mice were generated by reconstituting lethally-irradiated: (i) BALB/c recipients with BALB/c BM (green bars), (ii) BALB/c recipients with *Chi3l1*$^{-/-}$ BM (blue bars), (iii) *Chi3l1*$^{-/-}$ recipients with BALB/c BM (yellow bars) and (iv) *Chi3l1*$^{-/-}$ recipients with *Chi3l1*$^{-/-}$ BM (red bars). 8 weeks after reconstitution mice were infected with *Hp*, then msLN cells were analyzed 8 days post-infection directly *ex vivo* (**A-H**) or following restimulation for 4 hours with anti-CD3 in the presence of BFA (**I-K**). Numbers of (**A**) msLN cells, (**B**) msLN $CD19^+$ B cells, (**C**) msLN $CD4^+$ cells, and (**D**) msLN $CD4^+$ $CD62L^{lo}CD44^{hi}$ cells from reciprocal BM chimeras (n=8-10 mice/group) were enumerated on D8 post-*Hp* infection. Representative flow plots (**E**) showing $CXCR5^+PD\text{-}1^{hi}$ T_{FH} cells with the frequency (**F**) and number (**G**) of msLN T_{FH} cells in each group. gMFI of ICOS staining (**H**) by T_{FH} cells in each of the 4 groups of chimeras. (**I-K**) IL-4 and IL-13 production by resting and anti-CD3 stimulated msLN $CD44^{hi}$ CD4 cells (**I**) with the number of $IL\text{-}4^+IL\text{-}13^+$ (**J**) and total $IL\text{-}4^+$ (**K**) producers. Data representative of cumulation of 2 experiments repeated twice (n=4-5 mice per group per experiment), displayed as the mean±SD of each group with cells from individual animals depicted as circles or triangles. One-way ANOVA was used to assess statistical significance. Significant P values: (**D**) $p < 0.05$, (**F**) $p < 0.0001$, (**G**) $p < 0.01$, (**H**) $p < 0.01$, (**J**) $p < 0.01$, (**K**) $p < 0.0001$, (**L**) $p < 0.001$, (**M**) $p < 0.01$. Post-hoc analysis significance denoted on graphs: *p≤0.05, **p≤0.01, ***p≤0.001, ****p≤0.0001.

Supplementary Material

Chitinase-3-like 1 regulates T_H2 cells, T_{FH} cells and IgE responses to helminth infection

Miranda L. Curtiss[1*], Alexander F. Rosenberg[2,3], Christopher D. Scharer[4], Betty Mousseau[2], Natalia A. Ballesteros Benavides[1,2], John E. Bradley[5], Beatriz León[2], Chad Steele[6], Troy D. Randall[5], Frances E. Lund[2]

*** Correspondence:** Corresponding Author: mlcurtiss@uabmc.edu

Supplementary Figure 4

Supplemental Figure S4 showing supporting information for Figure 3. *Chi3l1* regulates B cell development in the BM and spleen via a cell intrinsic mechanism. (**A-F**) Analysis of donor-derived splenic T cells, B cells and myeloid cells in uninfected 50:50 BALB/c (CD45.1$^+$) and *Chi3l1*$^{-/-}$ (CD45.2$^+$) BM chimeras. Radiation chimeras were generated by reconstituting lethally-irradiated BALB/c recipient mice with a mixture of (**B**) of BALB/c (CD45.1$^+$, teal bars) and *Chi3l1*$^{-/-}$ (CD45.2$^+$, orange bars) bone marrow (BM), with representative flow of input BM shown in (**A**). Uninfected mice (n=5/group) were analyzed at 8 weeks post-reconstitution. (**C-F**) Representative splenocyte flow plots identifying CD19$^+$ cells, CD4$^+$ cells, CD8$^+$ T cells, and CD3$^-$CD19$^-$DX5$^-$CD45$^+$ myeloid cells (**C**) that were then subdivided using the CD45 congenic marker (**D**) to identify BALB/c and *Chi3l1*$^{-/-}$ cells. The percentage of CD45.1$^+$ BALB/c cells within the CD19$^+$ B, CD4$^+$ T, CD8$^+$ T, and CD45$^+$CD19$^-$CD3$^-$NK$^-$ splenocytes (**E**) is shown. The percentage of CD45.1$^+$ BALB/c cells within the CD19$^+$ B, CD4$^+$ T, CD8$^+$ T, and CD45$^+$CD19$^-$CD3$^-$NK$^-$ splenocytes after normalizing for input BM (**F**) is shown. (**G-K**) Flow plots showing gating of BM fractions A-C' (**G**) and fractions D-F (**I**) with flow plots showing percentages of BALB/c (CD45.1$^+$, teal bars) and *Chi3l1*$^{-/-}$ (CD45.2$^+$, orange bars) donor-derived developing B cells in fractions A-C' (**H**) and fractions D-F (**J**). Cells were pre-gated on lineage markers (negative for CD3, CD11b, Ter-119, Gr-1, Ly-6G, and DX5). The percentage of CD45.1$^+$ BALB/c cells within the BM B cell subsets after normalizing for input BM (**K**) is shown. (**L-N**) Flow plots showing gating of spleen B cells into follicular (FO) and marginal zone (MZ) B cell populations (**L**) with flow plots showing percentages of BALB/c (CD45.1$^+$, teal) and *Chi3l1*$^{-/-}$ (CD45.2$^+$, orange) donor-derived B cells (**M**). The percentage of CD45.1$^+$ BALB/c cells within the MZ and FO B cell

subsets after normalizing for input BM (**N**) is shown. Data representative of ≥3 independent experiments. Data displayed as the mean±SD in triplicate shown as bars (**B**) or as mean±SD of each group with individual animals (**E, F, K, N**) depicted as circles or triangles. Normalization (normalized $CD45^+$, open) for each animal (**F, K, N**) was performed against mean of input wildtype BM analyzed in triplicate (see panel **B**). Statistical analysis was performed with unpaired 2-tailed student's t-test (**B**), 1-way ANOVA (**E**, **F, N**) or 1-way ANOVA against Fr A developing B cells (**K**). *p≤0.05, **p≤0.01, ***p≤0.001, ****p≤0.0001. *p≤0.05, **p≤0.01, ***p≤0.001, ****p≤0.0001.

Supplementary Material

Chitinase-3-like 1 regulates T_H2 cells, T_{FH} cells and IgE responses to helminth infection

Miranda L. Curtiss[1*], Alexander F. Rosenberg[2,3], Christopher D. Scharer[4], Betty Mousseau[2], Natalia A. Ballesteros Benavides[1,2], John E. Bradley[5], Beatriz León[2], Chad Steele[6], Troy D. Randall[5], Frances E. Lund[2]

*** Correspondence:** Corresponding Author: mlcurtiss@uabmc.edu

Supplementary Figure 5

Supplemental Figure S5 showing supporting information for Figure 5. Germinal center reactions in msLN of D14 *Hp*-infected mice. Characterization of B cell responses in msLNs of uninfected (n=5/group) and D14 *Hp*-infected (n=7/group) BALB/c and *Chi3l1*$^{-/-}$ mice. (**A-C**) Representative B cell subset flow plots from BALB/c and *Chi3l1*$^{-/-}$ mice with gating strategies to identify (**A**) B220^{lo}CD138^{hi} ASCs (blue gate), (**B**) IgD^{neg}IgM^{neg} isotype-switched B cells (purple gate) and naïve B cells (cerulean gate) and (**C**) PNA^{hi}CD38^{lo} GCB cells (green gate). (**D-F**) Enumeration of total msLN cells (**D**), B220^{+}CD138^{-} B cells (**E**) and naïve B cells (**F**) in uninfected recipients and *Hp*-infected recipients 14 days post-infection (**G-H**). Gating analysis of PNA expression on IgD^{neg}IgM^{neg} isotype-switched B cells from uninfected or D14 *Hp*-infected wildtype msLN. **G.** Gates for CD38^{+}PNA^{-} (black gate), CD38^{int}PNA^{+} (teal gate), CD38^{lo}PNA^{+} (blue gate) and GC B gates (green gate). Including CD38$^{int/lo}$PNA^{+} msLN cells. **H.** PNA staining levels by each gated population shown in (**G**) with matching color histogram relative to FMO (gray histogram). Data representative of ≥3 independent experiments. Data displayed as the mean±SD in triplicate shown as bars. Statistical analysis was performed with unpaired 2-tailed student's t-test. *p≤0.05, **p≤0.01, ***p≤0.001, ****p≤0.0001.

Supplementary Material

Chitinase-3-like 1 regulates T_H2 cells, T_{FH} cells and IgE responses to helminth infection

Miranda L. Curtiss[1*], Alexander F. Rosenberg[2,3], Christopher D. Scharer[4], Betty Mousseau[2], Natalia A. Ballesteros Benavides[1,2], John E. Bradley[5], Beatriz León[2], Chad Steele[6], Troy D. Randall[5], Frances E. Lund[2]

*** Correspondence:** Corresponding Author: mlcurtiss@uabmc.edu

Supplementary Figure 6

Supplemental Figure S6 showing supporting information for Figure 5. Analysis of B cell responses following *Hp* infection. Analysis of B cells in *Hp*-infected 50:50 BALB/c (CD45.1$^+$) and *Chi3l1*$^{-/-}$ (CD45.2$^+$) BM chimeras. **A.** Radiation chimeras were generated by reconstituting lethally-irradiated BALB/c recipient mice with a 1:1 mix of BALB/c (CD45.1$^+$) and *Chi3l1*$^{-/-}$ (CD45.2$^+$) BM. (**B-E**) Representative gating of CD138$^+$B220$^{lo/int}$ ASCs and B220$^+$CD138$^-$ B cells (**B**) within the BALB/c (CD45.1$^+$) or *Chi3l1*$^{-/-}$ (CD45.2$^+$) CD4^{neg} msLNs from uninfected and D14 *Hp*-infected mice. Frequencies of ASCs of each genotype (**C**) derived from each animal at D0 and D14 post-*Hp* infection, with the percentage of BALB/c (CD45.1$^+$, teal circles) and *Chi3l1*$^{-/-}$ (CD45.2$^+$, orange circles). Representative gating (**D**) and frequencies (**E**) of each genotype of B220$^+$CD138$^-$ B cells in uninfected and D14 *Hp*-infected mice. BALB/c (CD45.1$^+$, teal gate) and *Chi3l1*$^{-/-}$ (CD45.2$^+$, orange gate). (**F-K**) Representative gating of PNA$^+$B220$^+$ GC B cells (**F**) within the BALB/c (CD45.1$^+$) or *Chi3l1*$^{-/-}$ (CD45.2$^+$) B cell population in uninfected and D14 *Hp*-infected mice. Frequencies of GCB cells of each genotype derived from each animal at D0 (**G**) and D14 (**H**) post-*Hp* infection from individual animals is shown as paired lines. (**I-J**) Representative gating showing the frequency of PNA$^+$B220$^+$ cells from uninfected and D14 *Hp*-infected mice (**I**). Representative gating (**J**) and frequencies (**K**) of each genotype of PNA$^+$B220$^+$ GC B cells derived from individual animals, shown as paired lines. (**L-N**) Analysis of IgE$^+$ ASCs in D14 *Hp*-infected mice. Representative flow plot (**L**) of D14 *Hp*-infected msLN CD138$^+$B220$^{lo/int}$ ASC, with the percentage of BALB/c (CD45.1$^+$, teal circles) and *Chi3l1*$^{-/-}$ (CD45.2$^+$, orange circles). ASCs from each genotype were analyzed for expression of IgE with a representative flow plot from a single animal shown (**M**). Data (**N**) analyzed as the % IgE$^+$ ASCs within

the ASC compartment of each genotype from infected animals and are shown as paired lines. Data representative of 2 independent experiments. Statistical analysis was performed with paired 2-tailed student's t-test. *$p \leq 0.05$, **$p \leq 0.01$, ***$p \leq 0.001$, ****$p \leq 0.0001$. *$p \leq 0.05$, **$p \leq 0.01$, ***$p \leq 0.001$, ****$p \leq 0.0001$

Supplementary Material

Chitinase-3-like 1 regulates T_H2 cells, T_{FH} cells and IgE responses to helminth infection

Miranda L. Curtiss[1*], Alexander F. Rosenberg[2,3], Christopher D. Scharer[4], Betty Mousseau[2], Natalia A. Ballesteros Benavides[1,2], John E. Bradley[5], Beatriz León[2], Chad Steele[6], Troy D. Randall[5], Frances E. Lund[2]

*** Correspondence:** Corresponding Author: mlcurtiss@uabmc.edu

Supplementary Figure 7

Supplemental Figure S7 showing supporting information for Figure 6. Germinal center reactions in spleens of D12 NS1/alum-immunized mice. Characterization of B cell responses in spleens of unimmunized control mice (n=5/group), immunized (n=5-6/group) BALB/c and *Chi3l1*$^{-/-}$ mice 12 days after *i.p.* injection with recombinant NS1 protein adsorbed to alum, and BALB/c mice 14 days after infection with *Hp* L3 larvae. (**A-C**) Representative B cell subset flow plots from BALB/c and *Chi3l1*$^{-/-}$ mice with gating strategies to identify (**A**) B220^{lo}CD138^{hi} ASCs (blue gate) and B220^{+}CD138^{-} B cells (red gate), (**B**) IgD^{neg}IgM^{neg} isotype-switched B cells (purple gate) and naïve B cells (cerulean gate) and (**C**) PNA^{hi}CD38^{lo} GCB cells (green gate). (**D-I**) Enumeration of total splenocytes (**D**) and B220^{lo}CD138^{hi} ASCs (**E**) from control mice or mice 12 days after immunization. Percentage (**F**) and number (**G**) of IgD^{neg}IgM^{neg} isotype-switched B cells, percentage (**H**) and number (**I**) of PNA^{hi}CD38^{lo} GCB cells from control mice or mice 12 days after immunization. (**J-M**) Representative gating to identify NS1 tetramer-binding IgD^{neg}IgM^{neg} isotype-switched B cells (**J**) and PNA^{hi}CD38^{lo} GCB cells (**L**). Percentages of NS1 tetramer-binding IgD^{neg}IgM^{neg} isotype-switched B cells (**K**) and PNA^{hi}CD38^{lo} GCB cells (**M**) from control mice or mice 12 days after immunization. Cells from D14 *Hp*-infected mice shown as a negative control for NS1 staining. Data representative of $\geq$ 2 independent experiments. Data displayed as the mean±SD of each group with individual animals depicted as circles or triangles. Statistical analysis was performed with unpaired 2-

tailed student's t-test between control BALB/c and *Chi3l1*$^{-/-}$ mice or between NS1-immunized BALB/c and *Chi3l1*$^{-/-}$ mice. Statistical analysis in **K** amd **M** was performed only on NS1-immunized mice as NS1^{+} cells were too low to be detected in unimmunized control mice. *p≤0.05, **p≤0.01, ***p≤0.001, ****p≤0.0001.
